# Titania-Based Hybrid Materials with ZnO, ZrO_2_ and MoS_2_: A Review

**DOI:** 10.3390/ma11112295

**Published:** 2018-11-15

**Authors:** Adam Kubiak, Katarzyna Siwińska-Ciesielczyk, Teofil Jesionowski

**Affiliations:** Institute of Chemical Technology and Engineering, Faculty of Chemical Technology, Poznan University of Technology, Berdychowo 4, PL-60965 Poznan, Poland; adam.l.kubiak@doctorate.put.poznan.pl

**Keywords:** titanium dioxide, zinc oxide, zirconia, molybdenum disulfide, binary systems, hybrid materials

## Abstract

Titania has properties that enable it to be used in a variety of applications, including self-cleaning surfaces, air and water purification systems, hydrogen evolution, and photoelectrochemical conversion. In order to improve the properties of titanium dioxide, modifications are made to obtain oxide/hybrid systems that are intended to have the properties of both components. In particular, zinc oxide, zirconia and molybdenum disulfide have been proposed as the second component of binary systems due to their antibacterial, electrochemical and photocatalytic properties. This paper presents a review of the current state of knowledge on the synthesis and practical utility of TiO_2_-ZnO and TiO_2_-ZrO_2_ oxide systems and TiO_2_-MoS_2_ hybrid materials. The first part focuses on the hydrothermal method; then a review is made of the literature on the synthesis of the aforementioned materials using the sol-gel method. In the last section, the literature on the electrospinning method of synthesis is reviewed. The most significant physico-chemical, structural and dispersive-morphological properties of binary hybrid systems based on TiO_2_ are described. A key aim of this review is to indicate the properties of TiO_2_-ZnO, TiO_2_-ZrO_2_ and TiO_2_-MoS_2_ hybrid systems that have the greatest importance for practical applications. The variety of utilities of titania-based hybrid materials is emphasized.

## 1. Introduction

Constant scientific and technological progress, as well as the desire to create environment-friendly technologies, is leading to intensive work on obtaining new-generation, functional products with strictly designed physico-chemical and dispersive-morphological properties, dedicated to specific applications. These studies focus on changing the physico-chemical features of many well-known and widely used materials. This group of substances includes oxide and hybrid materials based on titanium dioxide, which are distinguished by specific, precisely defined physico-chemical and structural properties, determined chiefly at the stage of their synthesis. Research on the synthesis of advanced materials based on titanium dioxide is becoming oriented towards the conscious and skilled modification of those materials’ properties, achieved by selecting appropriate methods and process conditions. For every method, the selection of conditions such as the precursors used, the implementation of the process, the final processing temperature and the pH of the reaction system has a decisive impact on properties of the product such as its dispersive character, morphology, thermal and colloidal stability, parameters of porous structure, and hydrophilicity or hydrophobicity. The wide range of possible methods of synthesis enables the design of materials of this type with diverse physico-chemical and structural parameters, and is crucial in view of the constant demand for such hybrids. Furthermore, technological progress is accompanied by increased interest in and development of methods enabling better control of the process, and thus also of the properties of the synthesized materials. 

Metal oxide systems have attracted a great deal of attention in recent years on account of their special electronic and chemical properties. Among the metal oxide semiconductors, compounds such as TiO_2_, ZrO_2_ and ZnO have been investigated extensively due to their chemical stability and good photocatalytic properties [1]. Titanium dioxide is the most widely used metal oxide for environmental applications, paints, electronic devices [2], gas sensors [3] and solar cells [4,5]. It is a well-known semiconductor with excellent photocatalytic properties, which has been widely used in environmental pollutant elimination [6,7], antibacterial dopes, self-cleaning surface, etc. [8]. Its unique antibacterial properties make it a candidate for applications in medical devices and sanitary ware surfaces [9]. Moreover, titania incorporated into polyester fabric [10,11] and related materials can be used as an absorber of harmful UV irradiation. Zinc oxide, due to its antibacterial [12] and photocatalytic activity [13], as well as its wide bandgap, has found applications in various areas based on its optical [14], piezoelectric and gas sensing properties [15,16], and in addition zinc compounds have generally been regarded as safe [1,17]. Zirconia is one of the most intensively studied materials owing to its technologically important applications in gas sensors [18,19], fuel cell electrolytes [20], catalysts [21] and catalytic supports [22], metal oxide-semiconductor devices [23], and in view of its superior thermal and chemical stability [24] and other properties [25]. 

Among synthetic hybrid oxide systems, particular attention is given to TiO_2_-ZrO_2_ materials, which thanks to the addition of zirconium dioxide have much greater surface area and mechanical strength than pure TiO_2_. Conjugation of zirconia together with titania leads to obtain products which are characterized with higher specific surface in comparison to pristine TiO_2_. Moreover, the addition of ZrO_2_ to TiO_2_ inhibits phase transformation of anatase-to-rutile, and creates more active site groups on titania surfaces [22,24]. There are many publications concerning the application of TiO_2_-ZrO_2_ hybrids in photocatalysis [26,27]. The use of TiO_2_-ZrO_2_ hybrid materials in the photo-oxidation of organic compounds or degradation of dyes originating from various industrial plants is well known. They also have applications in the photo-reduction of atmospherically harmful oxides, like CO_2_ and NOx, resulting for example from the combustion of fossil fuels. The advantages of the TiO_2_-ZrO_2_ hybrid are its mechanical strength, non-toxicity and corrosion resistance, and the ability to conduct photocatalytic processes using sunlight. These may be causes of increasing demand for this material in the near future [28,29,30]. 

Combining Titania with zinc oxide can also lead to a hybrid oxide system with good photocatalytic properties [31]. The resulting material can be used, for instance, in the degradation of organic impurities such as detergents, dyes and pesticides present in various types of wastewater. A TiO_2_-ZnO hybrid material can be synthesized by both physical and chemical processes, which enables enhancement of its properties, for example by widening its spectrum of light absorption. Additionally, the photocatalytic activity of oxides may help reduce the tendency of pollutants to form aggregate structures [32]. 

The combination of TiO_2_ with a semiconductor, such as molybdenum disulfide, allows the creation of a hybrid system that not only exhibits activity under the influence of visible radiation, but also allows the separation of photogenerated electron-hole pairs, which increases the catalytic capacity of the material [33,34].

The presented review focuses on recent stage on knowledge about sol-gel, hydrothermal and electrospinning synthesis of advanced, multifunctional materials based on titanium dioxide with strictly defined physico-chemical and structural properties, which significantly determine their multidirectional application. It is known that different properties of hybrid systems, such as TiO_2_-ZnO, TiO_2_-ZrO_2_ and TiO_2_-MoS_2_, depend on their morphology, crystallites size, and crystalline structure, which can be modified by selecting the appropriate method for their synthesis, as well as selecting the right components which together with TiO_2_ will create advanced hybrid materials with unique properties. 

## 2. Method of Synthesis of Titania-Based Materials

### 2.1. The Hydrothermal Method

One of the most frequently used methods for obtaining powder materials with specific physico-chemical and dispersive-morphological properties is hydrothermal synthesis. This can be used to obtain oxides such as TiO_2_, ZrO_2_, Al_2_O_3_, etc. According to the definition proposed by Roy [35] and Rubenau [36], hydrothermal synthesis is a reaction that takes place in a water environment at elevated temperature (>100 °C) and pressure, carried out in an autoclave [37,38].

**Figure 1:**
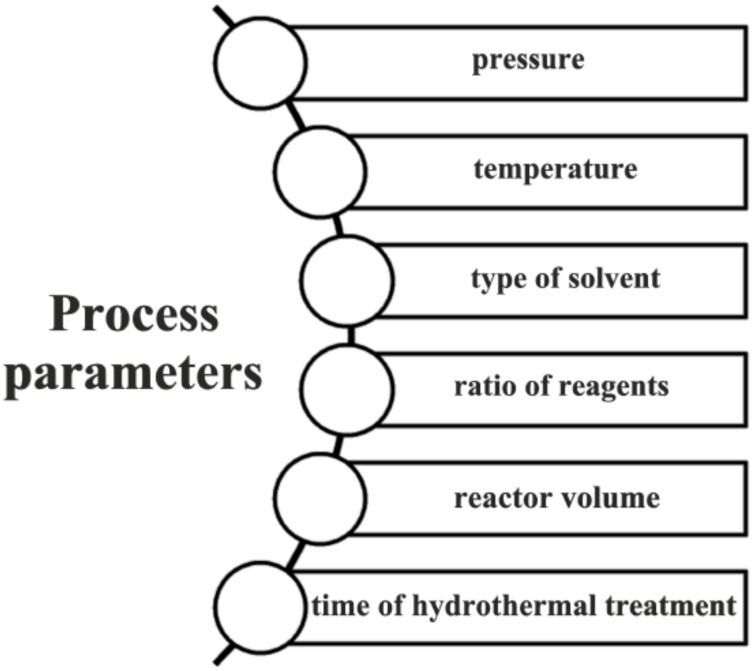
Process parameters determining the physico-chemical and structural properties of products obtained by the hydrothermal method.

The physico-chemical properties of products formed using the hydrothermal method depend on many process parameters, including the temperature, pressure, time of reaction, reactor volume, type of solvent used, and ratio of reagents (Figure 1). Literature data demonstrate clearly that the temperature and time of hydrothermal treatment [39,40], as well as the relative quantities of reagents used, have an effect on the crystal structure [41,42] and crystallite size of the synthesized material. Another important parameter is the reactor volume; this conditions the creation of (hydrostatic) pressure, which has a direct impact on the size of the synthesized particles [43,44,45]. 

The hydrothermal method offers many advantages, including a fast reaction rate, the quality and purity of the synthesized products, and the obtaining of materials with a crystalline structure and a smaller particle size. The hydrothermal method can also be used to obtain products in intermediate oxidation states, such as chromium(IV) oxide, as well as metastable compounds such as tellurium iodide (Te_2_I) [39]. Furthermore, it is extremely advantageous both environmentally and economically, because of its relatively low energy costs. Compared with other well-known and widely used techniques in which the growth of crystals requires significant energy inputs (high temperature and pressure), the hydrothermal method offers conditions of crystallite growth similar to natural conditions. This is consistent with the principles of green chemistry [46,47,48]. 

Some unquestionable disadvantages of the hydrothermal method include the complexity and cost of the equipment (resulting from the need for the autoclaves to be resistant to high pressure). Moreover, this method does not permit direct observation of the process, and it requires the reagents used to be soluble in water, which serves as the process medium [49,50,51].

Despite these drawbacks, the hydrothermal method is often combined with other techniques (combined methods) to improve the physico-chemical properties of the synthesized materials. One such technique was described by Komarneni et al. [52] and Tompsett et al. [53], who introduced microwaves to the hydrothermal reactor, enabling it to be heated more rapidly to the required temperature. This enhancement of the hydrothermal process is made possible by the special properties of Teflon. That material has an optical gate which enables the passage of waves characteristic for the microwave range. The microwave-assisted hydrothermal method offers a high speed of crystallization (faster reaction kinetics) and leads to products with a very narrow particle size distribution and controlled morphology.

Due to the advantages of both conventional and microwave-assisted hydrothermal methods, in recent years these techniques have been used more frequently for synthesizing oxides and hybrid systems. To obtain oxide systems with diverse physico-chemical and structural properties, many researchers have used titanium dioxide (TiO_2_)—a compound commonly applied in many branches of industry—as a base for combination with zinc oxide (ZnO), zirconium dioxide (ZrO_2_) and molybdenum disulfide (MoS_2_) [54,55]. These materials offer good photocatalytic and electrochemical properties. For this reason, it is a problem of interest to synthesize oxide systems such as TiO_2_-ZnO, TiO_2_-ZrO_2_ as well as TiO_2_-MoS_2_ hybrid system using the principles of the conventional and microwave-assisted hydrothermal methods. Table 1 summarizes the most significant literature reports concerning the synthesis of TiO_2_-ZnO binary systems using the hydrothermal method, with or without the action of microwaves.

Many literature reports indicate that the most photo-active crystalline form of titanium dioxide is anatase [4,5]. However, its use limited by relatively large band gap 3.2 eV [6]. Therefore, the scientists have focused on modifying the physico-chemical properties TiO_2_ to improve its photocatalytic activity. Modification may be performed by formation hybrid materials [26,28,33]. Xu et al. [8] developed the synthesis of a TiO_2_-ZnO oxide system based on the principles of the sol-gel and hydrothermal methods. A key part of their work was the evaluation of the photocatalytic properties of the resulting materials in the decomposition of methyl orange (C.I. Basic Orange 10). In the first step, titanium tetrabutoxide, acetic acid and distilled water were mixed with zinc acetate, diethylamine (DEA) and ethanol, after which the solution underwent aging until it became a gel. In the second step the product was hydrothermally treated at different temperatures and for different times. X-ray diffractograms revealed the presence of characteristic diffraction reflections for anatase. Increasing the temperature of the hydrothermal treatment was shown not to affect the crystal structure of the final product. Scanning electron microscope (SEM) images showed that the TiO_2_-ZnO oxide systems contained particles with near-spherical shape and with a marked tendency to agglomerate. It was also found that an increase in the temperature of hydrothermal treatment, and the calcination process, did not significantly alter the morphology of the TiO_2_-ZnO oxide system. In photocatalytic tests, all of the synthesized materials demonstrated high photoactivity. The material subjected to hydrothermal treatment at 150 °C for 24 h and additional calcination at 350 °C for 2 h produced a 55% yield of methyl orange degradation after 3 h.

Cheng et al. [56] obtained TiO_2_-ZnO oxide systems which were used for energy conversion in dye-sensitized solar cells (DSSCs). The synthesis process used previously obtained TiO_2_ nanospheres, which were hydrothermally treated with zinc nitrate. The products were found to contain the crystal structure of anatase and wurtzite, and had the surface areas (which was determined by the multipoint Brunauere Emmette Teller method (BET)) of 348.8, 187.3 and 122.2 m^2^/g. It was shown that an increase in the molar fraction of ZnO in the synthesized materials led to increased intensity of the diffraction peaks corresponding to wurtzite, and deterioration of the porous structure parameters. The synthesized systems were used as photoanodes in DSSCs. The tests showed that the TiO_2_-ZnO oxide systems had better optical properties and higher energy conversion efficiency (8.78%) than pure titanium dioxide (6.79%).

Similar results to the aforementioned reported by Xu et al. [8] were obtained by Li [58], who observed the presence of characteristic diffraction peaks for ZnO, Ti_3_O_5_ and Ti, and found the materials to contain particles with a fusiform shape. They also demonstrated that the crystallinity of ZnO declined with an increase in the fraction of TiO_2_ in the final product. Tests of the photocatalytic activity of the TiO_2_-ZnO binary oxide system showed that it yielded complete degradation of methyl orange (C.I. Basic Orange 10) after 30 min. With pristine ZnO, by contrast, complete degradation occurred after 50 min.

With regard to the extensively described good electrochemical properties of TiO_2_ [67,68,69] and ZnO [70,71,72], Vlazan et al. [59] undertook work on the synthesis of a TiO_2_-ZnO oxide system in the form of core-shell nanoparticles, and went on to determine the electrochemical properties of the synthesized material. In the first stage of the synthesis, ZnO was obtained as a result of hydrothermal treatment of a mixture of zinc nitrate and a sodium hydroxide solution. The ZnO was then added to a suspension of poly(vinyl alcohol) and titanium tetraisopropoxide, and the reaction mixture was treated hydrothermally at 220 °C for 5 h. Analysis by techniques including X-ray diffraction (XRD) and scanning electron microscopy (SEM) showed that the obtained binary oxide system had the crystal structure of anatase and wurtzite, and that its particles were of spherical shape and measured 30 nm in diameter. Compared with the materials described by Cheng et al. [56], the obtained TiO_2_-ZnO oxide system had a smaller BET surface area (44.4 m^2^/g) and total pore volume (0.1132 cm^3^/g). The study also included an investigation of the electrochemical properties of the synthesized binary material. It was found that the electrical resistance of the TiO_2_-ZnO oxide system decreased with increasing temperature, from ρ = 1.2·× 10^8^ Ω (−128 °C) to ρ = 7.52·× 10^6^ Ω (29 °C). At the same time the electrical conductivity increased from σ = 3.78·× 10^−8^ S/cm (−128 °C) to σ = 6.04·× 10^−7^ S/cm (29 °C). The results demonstrate that the synthesized material offers good semiconducting properties.

The common use of titania in photocatalysis [54] motivated Zhang et al. [60] to test an oxide system based on TiO_2_ in a degradation process of C.I. (Colour Index) Basic Orange 10. Titanium tetrabutoxide and zinc acetate, dissolved in a poly(ethylene glycol) solution, were used as precursors. The obtained solutions were mixed and then treated hydrothermally at 120 °C for 6 h. To improve its crystallinity, the resulting oxide system was subjected to further hydrothermal treatment at 180–200 °C for a specified time. Based on X-ray diffraction results it was shown that the materials contained the crystal structure of anatase and wurtzite. It was observed that increasing the temperature and time of hydrothermal treatment improved the crystal structure of the analyzed materials. The TiO_2_-ZnO oxide systems contained particles of spherical shape with a tendency to agglomerate, and a range of sizes between 25 nm and 100 nm. The BET surface area was 206 m^2^/g and 97 m^2^/g respectively for materials treated at 180 °C and 200 °C. The synthesized materials were tested in the photocatalytic degradation of methyl orange (C.I. Basic Orange 10). The TiO_2_-ZnO oxide systems exhibited high activity in the degradation of that dye. The synthesized hybrids were also shown to have superior properties to those of pure TiO_2_ and ZnO.

**Figure 2:**
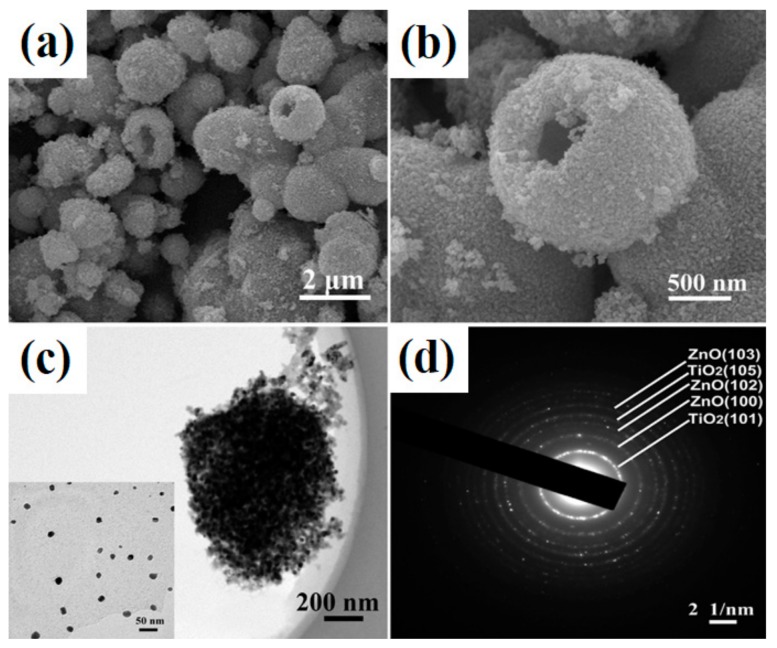
(**a**,**b**) SEM; (**c**) high-resolution transmission electron microscopy (HRTEM) and (**d**) selected area electron diffraction (SAED) images of the TiO_2_-ZnO system (created based on [61] with permission from Elsevier Publisher).

Spherical hollow structures are receiving a great deal of attention, because they offer the lowest surface/volume ratio for aggregated products. Recent literature reports indicate the high photocatalytic activity of this type of TiO_2_ [73,74] and ZnO [75] structures due to their large surface area, low density and highly efficient light-harvesting abilities. Wang et al. [61] showed that the use of titanium(IV) sulphate(VI) and zinc nitrate(V) as precursors in the hydrothermal synthesis of TiO_2_-ZnO binary materials enables the obtaining of products with a defined morphology, spherical hollow structures (Figure 2). The synthesized oxide systems were found to contain the crystal structure of anatase and wurtzite. It was confirmed that an increase in the time of hydrothermal treatment above 6 h did not significantly affect the crystal structure. Analysis of porous structure parameters showed the BET surface area to be equal to 55.9 m^2^/g. To confirm the high photocatalytic activity of the products, a test was conducted using the degradation of methyl orange (C.I. Basic Orange 10). The TiO_2_-ZnO oxide systems produced very high rates of dye degradation (total decoloration after 25 min in UV light and after 180 min in visible light).

Core-shell [76,77] nanoparticles have a core made of one material, coated with another material on top of it. In biological applications, core-shell nanoparticles have major advantages over simple nanoparticles, which can lead to improved antibacterial properties. Rusu et al. [62] developed a simple synthesis of a TiO_2_-ZnO oxide system in the form of core-shell structures, with an application in a process of bacterial degradation. In the first stage of the synthesis, ammonia water was added to a zinc acetate solution. Then titanium tetraisopropoxide was added dropwise to the solution, and the resulting reaction mixture was hydrothermally treated at 220 °C for 5 h. X-ray diffraction results showed the product to contain the crystal structure of TiO_2_ and ZnO. SEM images revealed particles of spherical shape with a tendency to agglomerate; the particle diameters were measured at 10–20 nm for TiO_2_ and 30–80 nm for ZnO. Based on the results of antibacterial and antifungal tests, it was found that the material had good biological properties. The TiO_2_-ZnO oxide system exhibited a biostimulating effect on the biosynthesis of fungi proteases, which is very important from the point of view of biotechnological applications. 

Methylene blue (C.I. Basic Blue 9) is the most commonly used substance for dyeing cotton, timber and silk. Therefore, it is of great importance to deal effectively with the pollution resulting from such processes [78,79]. Chen, Zhang, Hu and Li [63] proposed a simple synthesis of TiO_2_-ZnO binary oxide materials for use in methylene blue degradation. In the first stage of the synthesis, TiCl_4_ and ZnCl_2_ were dissolved in a water-ethanol mixture in the molar ratios Ti:Zn = 2:1, 1:1 and 1:2. Next, a urea solution was added with intense mixing for several hours, and then the mixture was subjected to hydrothermal treatment. The synthesized photocatalysts had similar crystal structure and morphology to the materials described in earlier studies. It was also observed that with an increase in the molar fraction of a given monoxide, there was an increase in the intensity of the diffraction peaks corresponding to its crystal structure. In the degradation of a model methylene blue solution, the synthesized TiO_2_-ZnO oxide systems demonstrated high photoactivity in the decomposition of the tested organic pollutant. Complete decoloration of the dye solution was observed after 180 min in the presence of the material obtained in the molar ratio TiO_2_:ZnO = 1:2.

The precisely defined crystal structure of this group of binary materials determines the range of their possible applications. In an investigation of the formation of specific mixed crystal structures, Wang et al. [64] carried out a synthesis of TiO_2_-ZnO oxide systems using the hydrothermal method, supported by an additional calcination process. Diffractograms showed that the materials obtained after the hydrothermal treatment had an amorphous structure, whereas the TiO_2_-ZnO oxide systems that had been calcined at 600, 700 and 900 °C showed the presence of diffraction reflections characteristic for the crystal structure of anatase (600, 700 °C), rutile (700, 900 °C), wurtzite, Zn_2_TiO_8_, Zn_2_TiO_4_ and ZnTiO_3_. To determine the temperature of transformation of specific crystal structures, differential thermal analysis (DTA) was conducted. It was proved that the endothermic peaks at 140, 250, 800 and 940 °C were associated with, respectively, ammonia decomposition, the transformation of Zn_2_TiO_8_ to ZnTiO_3_ and rutile, and the transformation of ZnTiO_3_ to Zn_2_TiO_4_ and rutile. Furthermore, two exothermic peaks were observed at 560 and 690 °C, indicating the creation of a Zn_2_TiO_8_ form and the formation of ZnTiO_3_ and rutile.

**Figure 3:**
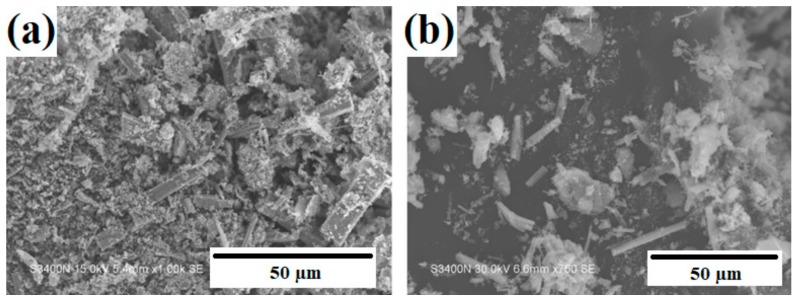
SEM images of a TiO_2_-ZnO oxide system subjected to calcination at temperatures of (**a**) 500 °C and (**b**) 600 °C (created based on [65] with permission from Elsevier Publisher).

The microwave hydrothermal method is a recently developed technique to prepare binary materials in very short times. The advantages of this process over the conventional hydrothermal method include extremely rapid kinetics of crystallization, very rapid heating to the treatment temperature, and the possible formation of new metastable phases [80,81]. Ashok, Venkateswara and Rao [65] developed a methodology for producing a TiO_2_-ZnO oxide system with the use of the hydrothermal method supported by the action of microwaves. In the first stage of the synthesis, a zinc acetate solution was added to titanium tetraisopropoxide, and then the mixture was alkalized with NaOH. The product was subjected to the action of microwaves for 5 min at 180 °C, using a frequency of 2.45 GHz. In the final step, the resulting oxide system underwent a calcination process in air at temperatures of 500 and 600 °C. X-ray analysis showed the presence of diffraction peaks corresponding to the crystal structure of anatase, wurtzite, and the zinc titanates (ZnTiO_3_ and Zn_2_Ti_3_O_4_). Images obtained by the SEM technique see (Figure 3) showed the TiO_2_-ZnO oxide systems to contain spherical particles with a tendency to agglomerate, irrespective of the temperature of thermal treatment. 

The synthesis of multi-component systems, despite its many advantages, such as the possibility of obtaining products with very good functional properties, is associated with certain problems, such as the need to optimize the process to obtain the best possible properties with substrates in given proportions. For this purpose, Divy et al. [66] used a combined method (a microwave-assisted hydrothermal process) to produce a TiO_2_-ZnO oxide system, which was then used as a matrix for a TiO_2_-ZnO-GO-Ag multi-component system. The matrix must have strictly defined parameters to produce a multi-component system with high photoactivity. For this reason, the synthesized TiO_2_-ZnO oxide system was analyzed with the use of techniques including XRD and BET. Diffractograms showed the presence of characteristic reflections for anatase and wurtzite. From low-temperature nitrogen sorption isotherms, the BET surface area was calculated to be 290 m^2^/g, the pore diameter 3.4 nm, and the pore volume 0.32 cm^3^/g. Because of the theoretically high photocatalytic activity of each component of the TiO_2_-ZnO-GO-Ag hybrid system, it was tested in the degradation of a model solution of rhodamine B (C.I. Basic Violet 10). The multi-component system exhibited high photoactivity (complete degradation of the dye after 60 min). It also demonstrated high stability, and could be used repeatedly over at least 5 catalytic cycles. 

In the next part of this review a survey will be made of work on TiO_2_-ZrO_2_ binary systems, synthesized—like the materials discussed in this section—using a hydrothermal process (for review see Table 2).

**Figure 4:**
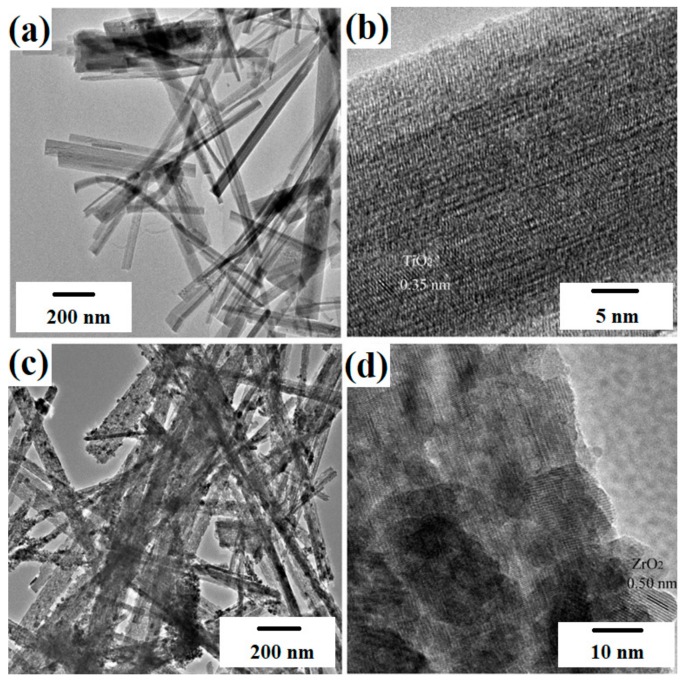
TEM images of (**a**) TiO_2_; (**b**) TiO_2_-ZrO_2_; HRTEM images of (**c**) TiO_2_ and (**d**) TiO_2_-ZrO_2_ (created based on [82] with permission from Elsevier Publisher).

In recent years, due to the increased emphasis on environmental protection, interest has grown in the use of biomass as a renewable source of fuel and organic chemicals, where fast or flash pyrolysis is used to produce bio-oils. In a study by Leahy [82], a TiO_2_-ZrO_2_ oxide material was obtained with the characteristic crystal structures of TiO_2_ and ZrO_2_. A scanning and transmission electron microscope (SEM and TEM) images (Figure 4) showed the deposition on nanorods of commercial TiO_2_ of ZrO_2_ particles with a diameter of approximately 20 nm. To enable the use of the synthesized oxide systems in the esterification of levulinic acid, surface modification with sulfate groups (SO_4_^2−^) was carried out. In tests of the extraction process, the TiO_2_-ZrO_2_ system attained a high yield of 70% (for a reaction taking place at 80 °C).

Semiconductors such as titania are very important materials with potential uses in diverse applications. A significant property of semiconductors is their electron gap [91], because a prerequisite for an efficient photocatalyst is that the redox potential for the evolution of hydrogen should lie within the band gap of the semiconductor. Tomar and Chakrabarty [83] described the synthesis of TiO_2_-ZrO_2_ oxide systems and determined their band gap energies. To obtain the oxide material, titanium tetraisopropoxide was mixed with zirconium tetraisopropoxide in the molar ratios TiO_2_:ZrO_2_ = 10:90, 30:70, 40:60, 60:40, 70:30 and 90:10, and the resulting reaction mixture was subjected to hydrothermal treatment at 240 °C for 24 h. The obtained materials were further enhanced by calcination at 450 °C for 4 h. Diffractograms revealed the crystal structures of anatase, rutile, and monoclinic and tetragonal ZrO_2_. The molar ratio used was found to affect the crystal structure: with an increase in the zirconia content, the diffractions peaks corresponding to ZrO_2_ became more intense. SEM images showed the particles to have a spherical shape. The band gaps, determined by the technique of UV-Vis spectroscopy, were measured at 2.48, 1.72, 1.34, 1.7, 1.49 and 1.49 eV respectively for the oxide systems synthesized in the molar ratios TiO_2_:ZrO_2_ = 10:90, 30:70, 40:60, 60:40, 70:30 and 90:10.

Kubo et al. [84] described the effect of a calcination process on the physico-chemical properties of a TiO_2_-ZrO_2_ oxide system, using zirconium tetraisopropoxide and titanium tetraisopropoxide. In the first stage, the precursors of TiO_2_ and ZrO_2_ were mixed in the molar ratios Zr:Ti = 1:0, 9:1, 6:1 and 3:1, and then underwent hydrothermal treatment at 300 °C for 2 h in a nitrogen atmosphere. After this process, the materials were subjected to additional calcination at 500 and 800 °C. X-ray diffraction analysis showed the presence of characteristic reflections for the crystal structure of tetragonal ZrO_2_. SEM images showed the synthesized materials to contain particles of spherical shape with a tendency to agglomerate. The effect of fusion of particles was also observed for the materials that had undergone thermal treatment (calcination). Low-temperature nitrogen sorption measurements gave BET surface areas of 200 and 80 m^2^/g respectively for the systems calcined at 500 and 800 °C. The conclusion of morphological and pore structure analysis was that an increase in calcination temperature caused a decrease in BET surface area.

**Figure 5:**
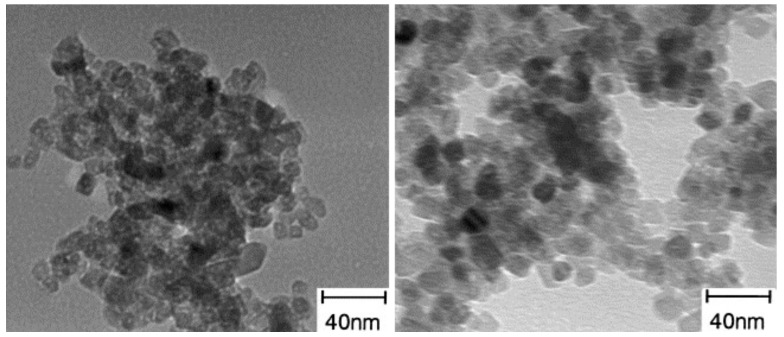
TEM images of the oxide system 90%TiO_2_-10%ZrO_2_ (created based on [85] with permission from Elsevier Publisher).

Many literature reports indicate the high photocatalytic activity of zirconia in the degradation of dyes such as methylene blue [92,93,94]. Hirano et al. [85] developed a simple hydrothermal synthesis of TiO_2_-ZrO_2_ binary oxide systems using titanium(IV) sulphate(VI) and zirconium sulphate(VI). Wide-ranging physico-chemical analysis confirmed the hydrothermal method as an effective process for the synthesis of binary oxide systems with defined crystallinity and morphology. The reported results indicate the presence of the crystal structure of anatase and monoclinic ZrO_2_. Transmission electron microscope images (Figure 5) showed that the particles of the TiO_2_-ZrO_2_ system had an almost spherical shape. In view of the presence of anatase and tetragonal ZrO_2_ in the synthesized material, an attempt was made to use the binary system in the process of degradation of methylene blue (C.I. Basic Blue 9). The TiO_2_-ZrO_2_ systems exhibited greater photocatalytic activity than the pure mono-oxides. The highest photoactivity in the degradation of the dye was achieved by the material obtained in the molar ratio TiO_2_:ZrO_2_ = 90:10.

At present the main focus in the field of DSSCs is the development of a photoanode that can efficiently harvest light [95]. Increased dye pickup, light scattering ability, reduced recombination reaction and improved charge transport ability are parameters that need to be engineered for high conversion efficiency. The properties of titania make it one of the most important DSSC photoanode materials. Tomar et al. [86] worked on a DSSC using a TiO_2_-ZrO_2_ system as the anode. In testing of the cell, the binary oxide material demonstrated a higher energy conversion efficiency (η = 1.97%) and fill factor (FF = 0.256) than pure TiO_2_ (η = 0.71%, FF = 0.061).

Yao et al. [87]—similarly to Hirano et al. [85]—described the use of a TiO_2_-ZrO_2_ oxide system in the process of photodegradation of methylene blue (C.I. Basic Blue 9). The material obtained in their study had a similar crystal structure to that described in [85]. SEM images revealed hollow microspheres of TiO_2_-ZrO_2_—this is closely linked to the high BET surface area of the material, which was determined from nitrogen adsorption/desorption isotherms to be 224 m^2^/g. Finally, the photocatalytic properties of the obtained binary material were tested in the degradation of methylene blue (C.I. Basic Blue 9). Use of the material led to a high level of degradation of the dye, reaching 99% after exposure to light for 60 min.

Caillot et al. [88] described the use of a synthesized TiO_2_-ZrO_2_ binary oxide system as an effective adsorbent of NH_3_ and SO_2_. The use of titanium(IV) and zirconium(IV) chlorides, and hydrothermal treatment at 220 °C, led to an amorphous structure. The absence of a defined crystal structure is linked to the high BET surface area (209 m^2^/g) and the unspecific particle shape. Results of energy-dispersive X-ray spectroscopy (EDX) indicated that the synthesized material contained 35% of zirconia and 65% of titania. Adsorption tests showed the material to have high adsorption capacities with respect to both NH_3_ and SO_2_; the values obtained were 573 µmol/g for NH_3_ and 414 µmol/g for SO_2_.

Ueda et al. [89] described the use of a titanium dioxide zirconium dioxide system to form bioinert foils. The synthesis of the material included hydrothermal treatment at 180 °C for 12 h. To enable use of the obtained materials in biological processes, the surface was modified using lactic acid. It was observed that while pure TiO_2_ had a high propensity to proliferate MC3T3-E1 cells (MC3T3 is an osteoblast precursor cell line derived from *Mus musculus* (mouse) calvaria), the synthesized oxide material showed a good ability to suppress proliferation of the cells, caused by a change in the surface morphology (increased roughness), which has a direct impact on the cell reproduction process. The experiment shows that the synthesized TiO_2_-ZrO_2_ material may be used for the purpose of slowing down osteointegration. The researchers also carried out a wide-ranging physico-chemical analysis to determine the morphological and crystal structure of the material. The synthesized binary oxide materials were found to contain the crystal structures of TiO_2_ and ZrO_2_, with the formation of a thin layer of ZrO_2_ on the surface of the TiO_2_. 

Kim et al. [90] synthesized a TiO_2_-ZrO_2_ system for use in a DSSC. In the first stage, the material was synthesized with the use of hydrothermal treatment at 260 °C for 12 h. X-ray diffractograms showed the presence of characteristic peaks for the anatase structure. The synthesized material was then used to prepare paste electrodes. Analysis of the porous structure of the electrode material showed it to have a BET surface area of 40.6 m^2^/g, a pore volume of 0.18 cm^3^/g and a pore diameter of 5.7 nm. In electrochemical tests, the material exhibited a high energy conversion efficiency of 2.88%, compared with 0.09% for commercial TiO_2_ (P25). 

The next part of this review will contain a survey of the literature on the synthesis and potential applications of TiO_2_-MoS_2_ hybrid systems (Table 3).

Indoles are among the most versatile and common nitrogen-based heterocyclic scaffolds and are frequently used in the synthesis of various organic compounds [106,107]. Indole-based compounds are very important among heterocyclic structures due to their biological and pharmaceutical activity. However, in order to improve their properties, it is necessary to modify them. Wang et al. [33] described a simple modification of indoles by thiocyanation, using a synthesized TiO_2_-MoS_2_ hybrid as a photocatalyst. Physico-chemical and structural analysis of this hybrid indicated a defined crystal structure and morphology. X-ray diffraction analysis showed the presence of reflections corresponding to the crystal structure of anatase TiO_2_ and MoS_2_. TEM images showed a layered structure of molybdenum disulfide with evenly distributed particles of titanium dioxide on the surface. Thiocyanation of indoles was carried out in a photocatalytic process, in which high yields were obtained. The highest photocatalytic activity (93%) was observed for the material synthesized in the molar ratio TiO_2_:MoS_2_ = 10:1.

Zhu et al. [34] synthesized a hybrid system containing the crystal structures of TiO_2_ and MoS_2_. Images obtained by scanning and transmission electron microscopy showed TiO_2_ particles uniformly distributed on the surface of the MoS_2_. Cyclic voltammetry curves showed the TiO_2_-MoS_2_ hybrid to have good electrochemical properties, with charge and discharge capacities at a current density of 100 mA/g equal to 643 and 827 mAh/g respectively in the first cycle, and 643 and 674 mAh/g in the second cycle. The capacity of the material also remained very stable over 100 subsequent cycles. It was concluded that the addition of anatase improved the electrochemical properties by facilitating the transport of electrons and ions during charging and discharging.

Ren et al. [96] used the hydrothermal method to obtain a TiO_2_-MoS_2_ hybrid material with strictly defined photoelectrochemical properties. Measurements showed the hybrids to have superior electrochemical properties to those of pure TiO_2_ or MoS_2_. The hybrid synthesized in the molar ratio TiO_2_:MoS_2_ = 20:1 had a photocurrent density of 33 µA/cm^2^ (compared with 8 µA/cm^2^ for TiO_2_). Photoelectrochemical results demonstrated a synergy effect between the titanium dioxide and molybdenum disulfide. A wide-ranging analysis of physico-chemical properties was also performed. The synthesized materials produced diffractions peaks corresponding to anatase and 2H-MoS_2_. For the hybrid obtained in the molar ratio TiO_2_:MoS_2_ = 20:1, the BET surface area was measured at 48.2 m^2^/g and the pore diameter at 7 nm. Morphological analysis confirmed that the particles of molybdenum disulfide were coated with particles of titanium dioxide.

Molybdenum disulfide photocatalysts are attracting wide attention as they offer a suitable band gap for visible-light harvesting, making the compound a promising earth-abundant photocatalyst for hydrogen production, environmental remediation, and photosynthesis [108,109]. A team of researchers [97] developed a two-stage method for obtaining active TiO_2_-MoS_2_ photocatalysts by hydrothermal synthesis. X-ray diffractograms revealed the crystal structure of TiO_2_ (for all of the materials) and MoS_2_ (only for the systems formed in the molar ratio Mo:Ti = 7.5%). The BET surface area was measured at 86, 87, 98 and 92 m^2^/g respectively for the systems with molar ratios Mo:Ti = 1%, 2.5%, 5% and 7.5%, and the respective band gaps were determined to be 3.15, 3.14, 3.1 and 2.97 eV. The synthesized TiO_2_-MoS_2_ hybrids were tested in a process of photodegradation of methylene blue (C.I. Basic Blue 9), and analysis of their photocatalytic activity showed that they produced high yields of degradation of the dye. For the system with Mo:Ti = 7.5% the solution was completely decolored after 60 min, indicating a tripling in the efficiency of photodegradation of the organic pollutant compared with pure TiO_2_. 

**Figure 6:**
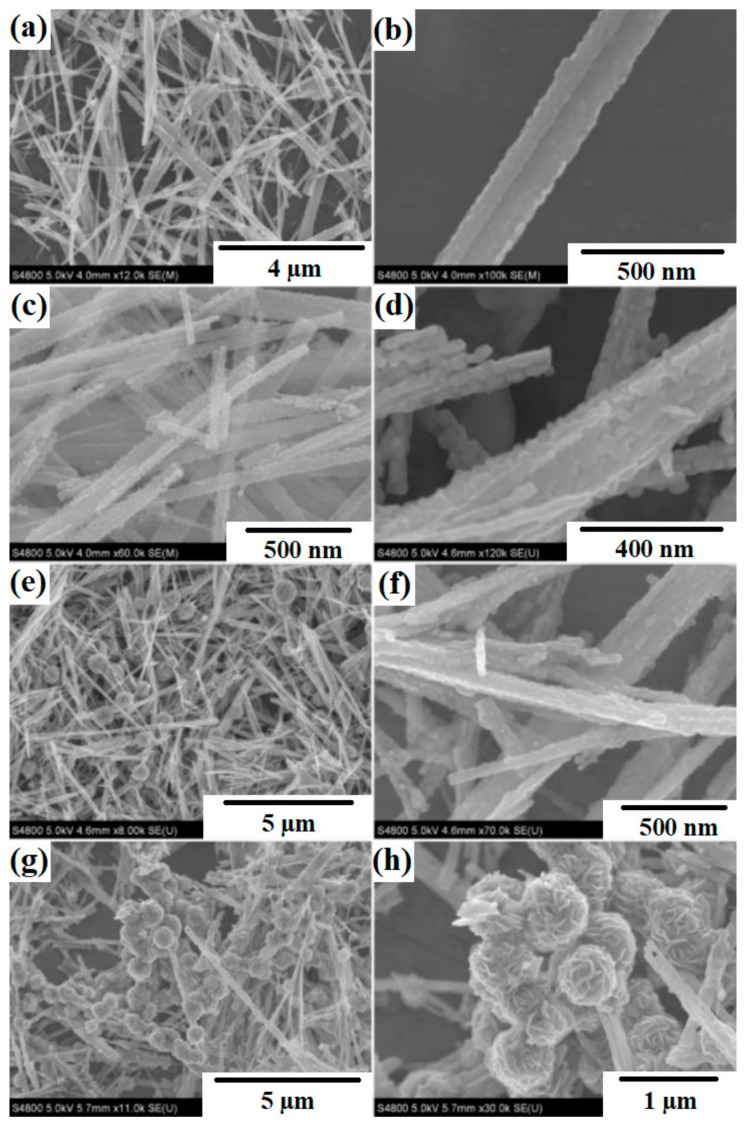
SEM images of TiO_2_-MoS_2_ hybrid systems with MoS_2_ contents of (**a**,**b**) 20%; (**c**,**d**) 40%; (**e**,**f**) 60%; and (**g**,**h**) 80% (created based on [98] with permission from Elsevier Publisher).

Liu et al. [98] described the synthesis of multifunctional TiO_2_-MoS_2_ hybrids with photoelectrochemical and photocatalytic properties. The substances used as precursors of TiO_2_ and MoS_2_ were commercial titanium dioxide (P25 Evonik) and sodium molybdenate. These were mixed to obtain the mass ratios TiO_2_:MoS_2_ = 80:20, 60:40, 40:60 and 20:80. The systems then underwent hydrothermal processing at 180 °C for 24 h. X-ray diffractograms contained characteristic peaks for TiO_2_, Ti_6_O_11_ and MoS_2_. SEM images (Figure 6) showed that spherical particles of titanium dioxide were deposited on nanofibers of molybdenum disulfide. For the resulting systems, a determination was made of their photoelectrochemical properties and photocatalytic activity in the removal of rhodamine B (C.I. Basic Violet 10). The TiO_2_-MoS_2_ hybrid system with the mass ratio TiO_2_:MoS_2_ = 60:40 exhibited high photocurrent density (12.5 µA/cm^2^). An increase in the content of molybdenum disulfide in the hybrid system led to a deterioration of its photoelectrochemical properties. In the photocatalytic degradation of rhodamine B, the highest photocatalytic activity was exhibited by the system with the mass ratio TiO_2_:MoS_2_ = 60:40. Complete decoloration of the dye solution was observed after 90 min.

Zhang et al. [99] described the use of TiO_2_-MoS_2_ binary hybrids in the photocatalytic degradation of methyl orange (C.I. Basic Orange 10). In the first stage, a two-step hydrothermal method was used to synthesize the aforementioned materials. TiO_2_ nanoparticles were formed by a sonication method. Molybdenum disulfide was then added to the TiO_2_, and the resulting mixture was subjected to hydrothermal treatment at 140 °C for 3 h. Unlike other previously described hybrids, the material obtained in this study was found to contain the crystal structure of anatase, ilsemanite and molybdenite. Scanning electron microscope images showed that spherical particles of titanium dioxide were deposited on MoS_2_ nanofibers. In photodegradation tests, the TiO_2_-MoS_2_ hybrid system exhibited high photocatalytic efficiency in the decomposition of a model dye solution after 60 min. 

Yang et al. [100] synthesized a hybrid system based on TiO_2_ and MoS_2_ using a hydrothermal method, with the aim of testing the resulting materials in a process of reduction of Cr(VI). In the first stage of synthesis, titanium dioxide was obtained from titanium in an anodic process. Next, thioacetamide and sodium molybdenate were dissolved in deionized water, the obtained titanium dioxide was added, and hydrothermal treatment was carried out at 220 °C for 24 h. X-ray diffractograms showed the synthesized materials to have the crystal structure of anatase and hexagonal MoS_2_. SEM images revealed hollow TiO_2_ nanotubes in which MoS_2_ particles were deposited. Photoelectrochemical tests demonstrated that the addition of MoS_2_ to the TiO_2_ structure reduced its resistance, leading to an improvement in the transport of electrons in the catalyst. A high yield of Cr(VI) reduction was obtained using the TiO_2_-MoS_2_ hybrid system, with a rate of reduction of 103.9 mg/L·min·cm^2^ at 0.1 V. Tests of the stability of the reaction process showed that the process efficiency did not deteriorate over five consecutive cycles.

**Figure 7:**
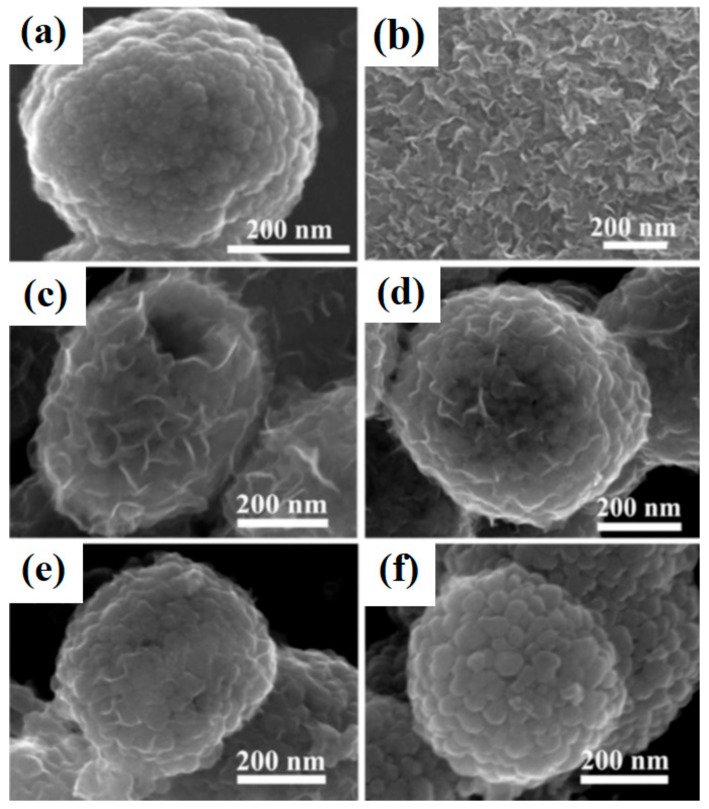
SEM images of (**a**) TiO_2_; (**b**) MoS_2_; (**c**) TM3; (**d**) TM6; (**e**) TM9 and (f) TM12 (created based on [101] with permission from Elsevier Publisher).

Phenol and its derivatives have been used in many industrial processes, including the production of dyes, polymers and medicines. Due to their high production volumes and widespread application, phenols and their derivatives are released into the environment from industrial and municipal liquid wastes [110,111]. This is particularly undesirable, since phenols pose a serious hazard due to their hematotoxic and hepatotoxic actions [112]. Wang et al. [101] described the use of synthesized TiO_2_-MoS_2_ hybrid systems in the process of photodegradation of phenol. At the first stage, hollow spheres of TiO_2_ were obtained [108,109]. Next, sodium molybdenate was added in the molar ratios TiO_2_:Na_2_MoO_4_ = 30:135 (sample TM3), 60:135 (sample TM6), 90:135 (sample TM9) and 120:135 (sample TM12). Diffractograms obtained for the TiO_2_-MoS_2_ materials contained characteristic peaks for the crystal structure of anatase TiO_2_ and rhombohedral MoS_2_. The anatase structure became less visible as the MoS_2_ content increased. Scanning electron microscope images (see Figure 7) showed particles of titanium dioxide with close to spherical shapes, hollow inside, with diameters of 600 nm, coated with a layer of molybdenum disulfide. The synthesized materials were tested in a process of photocatalytic degradation of phenol. The hybrids exhibited good photocatalytic properties: the yield of phenol degradation was 78% after 150 min in the presence of the system obtained in the mass ratio TiO_2_:Na_2_MoO_4_ = 90:135. Tests of photocatalytic stability showed that the photoactivity of this material did not decrease significantly over four consecutive catalytic cycles.

Bai et al. [104] were the first to report the combination of exfoliated MoS_2_ with TiO_2_ nanoparticles and the use of the resulting material in a photocatalytic process. To establish whether the materials could be successfully used as active photocatalysts, a comprehensive physico-chemical analysis was carried out. Recorded diffraction peaks corresponding to the crystal structure of TiO_2_ (anatase) and MoS_2_. Morphological parameters were investigated by the SEM and TEM techniques. The resulting images showed the particles of molybdenum disulfide to be densely covered with particles of titanium dioxide. Distances between layers were measured at 0.27 nm and 0.35 nm, which confirmed the presence of the structure of MoS_2_ and TiO_2_ nanocrystals. In tests of photocatalytic activity in the decomposition of rhodamine B (C.I. Basic Violet 10), 100% decomposition of the dye was attained after exposure to light for 30 min in the presence of the TiO_2_(anatase)-MoS_2_(1T) hybrid system, compared with 40% in the presence of the TiO_2_(anatase)-MoS_2_(2H) system. In addition, the TiO_2_(anatase)-MoS_2_(1T) system catalysed the evolution of hydrogen at a rate of 2 mmol/g·h, compared with 0.25 mmol/g·h for TiO_2_(anatase)-MoS_2_(2H) and 0.5 mmol/g·h for pure TiO_2_. 

Photocatalytic evolution of H_2_ over semiconducting materials is regarded as one of the promising solutions to the growing energy crisis, due to its potential application for clean hydrogen production from water [109,113,114]. Yuan et al. [105] described the use of a single-stage hydrothermal process to synthesize binary hybrid photocatalysts for the evolution of hydrogen. Photocatalytic hydrogen evolution experiments were mainly carried out in a Pyrex glass cell with a top window connected to a gas-closed system. The assessment of photocatalytic properties was carried out using 100 mg of H_2_-evolving photocatalyst powder which was suspended in 100 cm^3^ of an aqueous solution containing 10% methanol in volume. The resulting materials led to high yields of hydrogen: the highest—2200 µmol/g·h—was recorded for the system with a 0.5% MoS_2_ mass fraction (this is 36 times higher than the yield obtained with pure TiO_2_). For comparison, TiO_2_-Pt, TiO_2_-Pd, TiO_2_-Rh, TiO_2_-Au and TiO_2_-Ru were used as catalysts in the same process, giving yields of 1450, 1250 1000, 750 and 550 µmol/g·h respectively. Physico-chemical parameters were analysed by the XRD, TEM and BET techniques. X-ray diffractograms contained characteristic peaks for the crystal structure of anatase TiO_2_ and MoS_2_. Based on nitrogen sorption, a BET surface area of 105.6 m^2^/g was obtained. Transmission electron microscope images showed interplanar distances of 0.19 nm, characteristic for TiO_2_, and 0.62 nm, characteristic for hexagonal MoS_2_.

A comprehensive review of the literature concerning oxide systems containing titanium dioxide together with zinc oxide or zirconium dioxide, or TiO_2_-MoS_2_ hybrid systems, synthesized by the hydrothermal method confirms that by the suitable choice of process conditions (precursors, pH of the reaction system, molar ratio of reagents, temperature of thermal treatment) it is possible to achieve effective changes in the properties of the resulting materials. Appropriately selected process parameters make it possible to design the physico-chemical properties of synthesized oxide and hybrid materials, including particle shape and size, degree of crystallinity, phase and surface compositions, and porous structure. Moreover, hydrothermally synthesized materials of TiO_2_-ZnO, TiO_2_-ZrO_2_ and TiO_2_-MoS_2_ type are finding a wide range of applications in bacterial and fungal degradation and as photocatalysts in the decomposition of certain organic pollutants. Such systems are also used as electrode materials in lithium-ion cells and dye-sensitized solar cells.

### 2.2. The Sol-Gel Method

The consistent development of technology has led to increased awareness of the importance of chemical synthesis methods, particularly those involving the use of organic precursors. One of the basic types of synthesis in which metallo-organic precursors are used to synthesize binary hybrid systems is the sol-gel method [115,116,117,118,119,120]. In recent years many definitions of the sol-gel process have been put forward, including that of Dislich and Hinz [121], which covers only multicomponent oxides. According to the most popular and widespread definition, such a process is based on a hydrolysis reaction of metallo-organic precursors of metals (usually alkoxides) in a reaction mixture [31,122,123,124,125].

**Figure 8:**
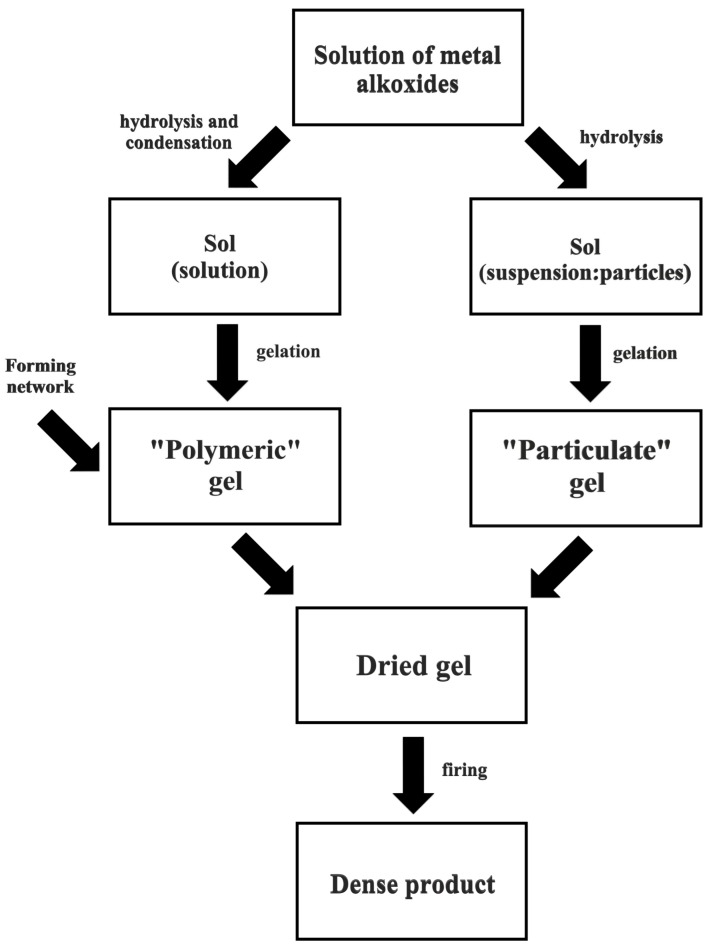
A block diagram of the sol-gel process (created based on [122] with permission from Springer).

A sol-gel process consists of several stages. In the first, a solution of metal alkoxides is mixed. This is followed by a process of hydrolysis and condensation, after which a sol is obtained [126,127,128,129,130,131,132]. In the next stage, gelation takes place—that is, the sol is transformed into a gel. The final stage involves the removal of residues of the liquid phase via a drying process. A block diagram of the sol-gel process appears in Figure 8 [133,134,135,136,137].

Sol-gel processes may be used in many branches of industry; for example, in the production of catalysts or catalyst supports [138], optical and electrical sensors [139], and implant coatings [139,140] Recent reports concern the use of the sol-gel method in the process of immobilization of a wide range of organic materials, including enzymes [141,142,143,144].

Compared with other conventional methods of synthesis, the sol-gel method has a number of advantages which enable its use in a wide range of technological processes. Some of these advantages are the ability to control the microstructure of the product, the use of low temperatures and the resulting low energy costs, the high homogeneity of the product, the absence of a need for complex apparatus, and the possibility of synthesizing new materials with designed properties [145,146].

The most significant drawbacks of the sol-gel process include the high costs of the metal precursors, the need to use reagents of high purity, the absence of total control over the process (the evolution of various oxides due to differences in the reactivity of the precursors), and the fusion of the product during calcination [122,138,139].

The sol-gel method may be used to obtain oxide systems containing TiO_2_. Titanium dioxide, because of its photocatalytic [147,148,149] and photoelectrochemical properties [150,151], represents an interesting base for the production of multifunctional oxide systems. There has recently been a marked growth in interest in such materials as TiO_2_-ZnO and TiO_2_-ZrO_2_ oxide systems, in view of the useful properties of zinc oxide (photocatalytic, photoelectrochemical and photoluminescent) and zirconium dioxide (photocatalytic and photoelectrochemical). 

In Table 4, an overview is given of the current stage of knowledge concerning the synthesis of TiO_2_-ZnO oxide systems using the sol-gel process. 

Stoyanova et al. investigated the effect of the type of precursor used, the TiO_2_:ZnO molar ratio and the calcination temperature on the physico-chemical properties of TiO_2_-ZnO oxide systems synthesized by the sol-gel method [1]. At the first stage, TiO_2_-ZnO systems were obtained using zinc acetate (sample A) and zinc chloride (sample B) as the ZnO precursor, and titanium tetrachloride as the precursor of TiO_2_. The TiO_2_:ZnO molar ratio was 90:10 (sample A) or 50:50 (sample B). The materials prepared from zinc acetate were calcined at 500 °C for 6 h, and those made using zinc chloride were calcined at 200, 400, 550 and 600 °C for 2 h. The diffractograms contained peaks corresponding to anatase in sample A, and to anatase, rutile and zinc titanate in sample B. It was demonstrated that the crystal structure of the final product was affected by the precursor type, the molar ratio of the reagents and the temperature of thermal processing. SEM images showed that the synthesized materials consisted of particles with a spherical shape and a marked tendency to agglomerate. Authors applied the obtained materials in a process of photodegradation of malachite green (C.I. Basic Green 4). They also determined the antibacterial properties of the materials against the Gram-positive *E. coli* (ATCC 25922). The results of the dye photodegradation experiment showed that both the material obtained from zinc acetate and that made using zinc chloride exhibited high photocatalytic activity: total degradation occurred after 40 min in the presence of sample B and after 170 min in the presence of sample A. In the tests with *E. coli*, the TiO_2_-ZnO system obtained using zinc chloride demonstrated 100% antibacterial activity after 120 min, while the system made using zinc acetate achieved only a 30% yield in the same amount of time. Under UV light, it was found that the bacteria were removed completely after 15 min in the presence of sample A and after 30 min in the presence of sample B. 

In Section 2.1 it was described how a TiO_2_-ZnO system synthesized by the hydrothermal method could be used in dye-sensitized solar cells [56]. Shanmugam et al. [152] also described the use of a binary oxide system in a DSSC, but applied the sol-gel method of synthesis. Electrochemical characterization of the cell showed it to have a current density of 13.1 mA/cm^3^ and a photoconversion efficiency of 32% (in darkness). The maximum equivalent quantum efficiency (EQE) of the DSSC built from a TiO_2_-ZnO oxide system was measured at 85%, compared with 67% for a cell based on TiO_2_ alone.

In recent years there have been reports concerning the potential use of non-toxic and inexpensive titania nanoparticles for imparting multifunctional properties to various textile materials [166,167,168]. Chu et al. [153] described the effect of the addition of a titania-based binary material on the antistatic properties of polyester fabrics. It was shown by physico-chemical analysis that the resulting materials had good stability in water, and that the stability improved as the molar fraction of ZnO was increased. SEM images of polyester fibres covered with TiO_2_-ZnO showed that the fabric was covered uniformly by a layer of the oxide material. The best antistatic properties were exhibited by materials obtained with TiO_2_:ZnO molar ratios of 5:5 and 7:3. Mechanical characterization of the materials showed that they offered greater permeability to gases than pure TiO_2_ or ZnO. A whiteness quality test showed that the white colouring of the obtained TiO_2_-ZnO systems was 10–30% lower than that of the reference samples.

Recent literature reports indicate that both titania [169,170] and zinc oxide [171] can be used as anti-corrosion coatings. Yu et al. [154] presented a simple synthesis of TiO_2_-ZnO oxide systems using the sol-gel method and described their properties as anti-corrosion coatings. In the first stage, TiO_2_ sol was obtained from titanium tetrabutoxide, ethanol, glacial acetic acid and water, and ZnO sol from zinc acetate, diethylamine, ethanol and water. These sols were mixed together in the molar ratios TiO_2_:ZnO = 0:1, 1:3, 1:1, 3:1, 1:0. The materials were applied to carbon steel using an immersion technique. The steel with the oxide layer was then dried at 70 °C for 10 h. Finally the materials were calcined at 350 and 500 °C for 2 h. The procedure was repeated two or four times to obtain coatings consisting of two, four or eight layers. The carbon steel plates coated with oxide materials were subjected to comprehensive physico-chemical analysis. The materials were shown to have the crystal structure of Fe, TiO_2_, Fe_3_O_4_, Fe_2_O_3_ and ZnFe_2_O_4_. X-ray diffractograms contained peaks originating from the iron present in the carbon steel. The material obtained at a TiO_2_:ZnO molar ratio of 1:0 was characterized by an anatase crystal structure. For the product calcined at 500 °C the forms Fe_3_O_4_ and Fe_2_O_3_ were observed, which indicates oxidation of the steel. SEM images showed that the materials calcined at 350 °C had numerous cracks, creating a non-uniform layer on the surface of the steel. An increase in the ZnO content in the oxide system was found to improve the uniformity of the coating. Potentiodynamic polarization curves were obtained to determine the anticorrosive properties of the coatings: the results showed that significant protection against corrosion was provided in contact with sea water. The best anticorrosive properties were obtained for the systems with TiO_2_:ZnO ratios of 1:1 and 1:3, thermally treated at 500 °C. 

To obtain a specific crystal structure of materials synthesized by the sol-gel method, it is necessary to carry out a calcination process. Many literature reports refer to the effect of calcination on photocatalytic properties [172,173,174]. Wang et al. [155] conducted a simple synthesis of a TiO_2_-ZnO oxide system using the sol-gel method and a calcination process. A TiO_2_ sol was obtained from titanium tetrabutoxide, anhydrous ethanol and hydrochloric acid, and a ZnO sol was obtained from zinc acetate, anhydrous ethanol and deionized water. Oxide systems were obtained by mixing the TiO_2_ and ZnO sols at the molar ratios ZnO:TiO_2_ = 0.1:0.15, 0.2:0.25, 0.3:0.35. In the final stage the oxide materials were calcined at 450, 480, 500, 550 and 600 °C for 1, 1.5, 2, 2.5 and 3 h. Since the physico-chemical properties of titania-based materials determine their photocatalytic ability, analysis was performed using such techniques as X-ray diffraction and scanning electron microscopy. The results of both XRD and SEM indicate the formation of the crystalline structure of anatase and wurtzite, and the spherical shape of the particles of the materials obtained. Their photocatalytic activity was evaluated with respect to the photodegradation of methyl orange (C.I. Basic Orange 10). All of the materials exhibited good degradation efficiency. The highest yield of dye degradation (93% after 5 h) was achieved by the material obtained at a ZnO:TiO_2_ molar ratio of 0.2:0.25.

Due to the increase in the number of cars and the emission of harmful gases in city centers, it is apparent that there is a need to remove pollutants such as nitrogen oxides (NO_x_). Many researchers, such as Dalton et al. [175], Karapati et al. [176] and Todorova et al. [177], have reported the use of titanium dioxide as an effective NO_x_ photocatalyst. Giannakopoulou et al. [156] described the synthesis of an effective binary oxide photocatalyst of nitrogen oxides using the sol-gel method. At the first stage of synthesis, TiO_2_ sol was obtained by mixing titanium tetrabutoxide with anhydrous ethanol, nitric acid and distilled water. The ZnO sol was produced using zinc acetate, ethylene glycol, glycerine, TEA and anhydrous ethanol. The sols were mixed together at the molar ratios TiO_2_:ZnO = 100:0, 75:25, 50:50, 25:75 and 0:100. Thin foils of the resulting binary oxide photocatalysts were produced using an immersion technique. The final step was calcination at 500 °C for 1 h. Analysis of X-ray diffractograms showed that the TiO_2_:ZnO molar ratio affected the parameters of the crystal structure. An increase in the ZnO content leads to the vanishing of the diffraction reflections characteristic of TiO_2_. The oxide materials with TiO_2_:ZnO molar ratios of 100:0 and 75:25 contained a single diffraction peak corresponding to anatase. When the ratio was 50:50 or 25:75 an amorphous structure was observed, while the system with 100% ZnO had the crystal structure of wurtzite. A test of photocatalytic activity showed that the highest activity in the oxidation of NO_x_ was achieved by the materials obtained with TiO_2_:ZnO molar ratios of 100:0 and 75:25. An increase in the content of ZnO led to a significant drop in photocatalytic activity, possibly due to the weak crystallinity of the materials obtained. 

**Figure 9:**
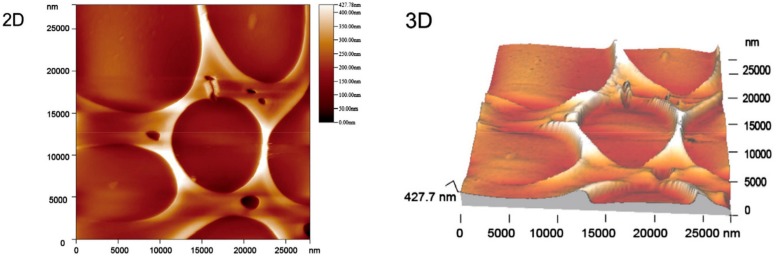
AFM images of the oxide system (90)TiO_2_-(10)ZnO (created based on [157] with permission from Elsevier Publisher).

The surface wettability of a material is one of the properties that determine its potential applications. Chen et al. [157] reported the synthesis of a superhydrophilic TiO_2_-ZnO oxide system in the form of a thin foil. It was produced using a TiO_2_ sol made from titanium tetrabutoxide, anhydrous ethanol, diethylamine (DEA) and nitric acid, and a ZnO sol obtained using zinc acetate, anhydrous ethanol and DEA. The precursor solutions were mixed together in the molar ratios TiO_2_:ZnO = 90:10, 80:20, 70:30, 60:40, and then underwent calcination at 500 °C for 30 min. The results relating to the crystal structure were similar to those reported by Giannakopoulou et al. [156]. An increase in the molar fraction of ZnO led to the vanishing of the anatase structure, and in the case of the materials with TiO_2_:ZnO ratios of 70:30 and 60:40, this resulted in the presence of an amorphous structure. Atomic force microscope (AFM) images (Figure 9) showed that the obtained composite foils had a microporous structure. Contact angle measurements showed all of the materials to be superhydrophilic, with contact angles in the range of 1.8–3.2° and diffusion of a drop of water on the surface of the material within 800 ms. A test involving the photodegradation of methylene blue (C.I. Basic Blue 9) showed that the addition of a small quantity (10%) of ZnO improved the material’s photocatalytic activity: the degradation yield was 80% after exposure to light for 280 min. 

Pournuroz et al. [158] analyzed the degradation of the dyes C.I. Reactive Red 195 (RR195) and C.I. Reactive Blue 19 (RB19) in the presence of a TiO_2_-ZnO oxide system. In the synthesis process sols of TiO_2_ and ZnO were obtained, which were mixed together in the molar ratio TiO_2_:ZnO = 10:3. The resulting material exhibited high photocatalytic activity. Complete degradation of the RR195 and RB19 dyes was observed after exposure to light for 40 and 60 min respectively, in the presence of the synthesized material.

The sol-gel auto-combustion synthesis method (also called low-temperature self-combustion, auto-ignition or self-propagation, as well as gel-thermal decomposition) combines the chemical sol-gel and combustion processes [178,179]. This method has shown great potential in the preparation of novel nanomaterials. Al-Mayman et al. [159] described the use of a sol-gel auto-ignition technique to synthesize a TiO_2_-ZnO oxide system with defined photocatalytic properties. At the first stage, TiO_2_ and ZnO precursors were dissolved in deionized water, and citric acid was added. The reaction mixture was then heated to 100 °C to evaporate off water. The resulting gel was ignited by heating to a temperature of approximately 300 °C. In the final stage, the TiO_2_-ZnO system was calcined in air at 500 °C for 5 h. Materials were obtained with the general formula Ti_1−x_Zn_x_O_2_, for x = 0%, 1%, 5%, 10%, 12% and 30%. Their diffractograms contained peaks corresponding to the crystal structure of anatase. Microstructural and morphological analysis indicated particles with a spherical shape and a marked tendency to agglomerate. The synthesized TO_2_-ZnO oxide materials had surface areas of 52, 63.4, 69.9, 64 and 36.6 m^2^/g when the mass fraction of ZnO was 1%, 5%, 10%, 12% and 30% respectively. The wide band gaps of titanium dioxide and zinc oxide make them promising materials for applications in the process of photocatalytic hydrogen production. All of the obtained materials were shown to be capable of evolving hydrogen under UV light. The highest yield was obtained from the material with the formula Ti_90_Zn_10_O_2_ (1.048 mmol/h of hydrogen after 9 h of irradiation).

Zulkiflee and Hussin [160] investigated the effect of the temperature of thermal processing on the physico-chemical properties of a TiO_2_-ZnO oxide system obtained by the sol-gel method. At the first stage a TiO_2_ sol was obtained from titanium tetrabutoxide, acetic acid and butanol, and a ZnO sol from zinc acetate, diethylamine and propan-2-ol. To obtain thin layers of the oxide material, glass plates were immersed in the titanium dioxide sol and calcined at 400 °C. The plates were then immersed in ZnO sol and calcined at 500 and 600 °C for 2 h. X-ray diffraction results showed that the materials obtained had the crystal structure of anatase and wurtzite. Atomic force microscope (AFM) observations demonstrated that an increase in the calcination temperature caused an increase in the roughness of the material surface. The band gaps of the synthesized materials were measured at 3.6 and 3.7 eV for the samples TiO_2_(400 °C)-ZnO(500 °C) and TiO_2_(400 °C)-ZnO(600 °C).

Prasannalakshmi and Shanmugam [161] used a TiO_2_-ZnO oxide system in the process of photocatalytic degradation of brilliant green (C.I. Basic Green 1) and methylene blue (C.I. Basic Blue 9). In the first stage of the synthesis, a TiO_2_ sol was obtained by hydrolysis of titanium tetraisopropoxide, and a ZnO sol was prepared from zinc acetate and 0.5 M sodium hydroxide. The sols were mixed together in the molar ratios TiO_2_:ZnO = 0.5:0.25, 1:0.5, 1.5:0.75, 2:1, to produce samples labelled TZO1 TZO2, TZO3 and TZO4. The final stage of the process was calcination at 550 °C for 4 h. In analysis of the crystal structure, all of the oxide systems gave diffraction reflections corresponding to anatase and wurtzite. The XRD results were confirmed using HRTEM analysis. HRTEM images showed distances between planes characteristic of (101) TiO_2_ and (101) ZnO. The surface areas, determined by low-temperature nitrogen adsorption and calculated based on BET theory, were 74.7, 32.2, 12.58 and 8.82 m^2^/g for the samples TZO1 TZO2, TZO3 and TZO4 respectively. All of the synthesized materials exhibited high photocatalytic activity, and each of the binary samples (TZO1, TZO2, TZO3, TZO4) had superior photocatalytic properties to those of pure TiO_2_ or ZnO. 

Materials such as titania [180,181] and zinc oxide [182] are successfully used for the construction of p-silicon heterojunction photodiodes. Al-Hazami and Yakuphanoglu [162] used a binary combination of these materials to build a p-type diode. A layer of the TiO_2_-ZnO oxide system was applied to a prepared silicone base, after which the material was dried and then calcined in air at 450 °C for 1 h. SEM results showed particles with a spherical shape with no tendency to agglomerate. The band gap was measured at 3.41 eV. Photoelectrochemical tests showed that the p-type diode covered with a thin foil of the TiO_2_-ZnO system had photovoltaic and photoconductive properties. Tests under different luminous intensities confirmed a rapid reaction to changes in the studied parameters and good process repeatability.

Taking account of the known antibacterial properties of titanium dioxide and zinc oxide, Armin et al. [163] carried out a wide-ranging analysis of possible applications of a TiO_2_-ZnO binary system. The obtained material was used in electrochemical, photocatalytic and antibacterial tests. Dynamic potentiometric curves showed that TiO_2_-ZnO coatings offered high resistance at low currents. The curves are contained within a very wide range of potentials, from −1.4 to 1.1 V. Based on the measured parameters, a corrosion index was computed for coated and uncoated materials. The material coated with TiO_2_-ZnO had a corrosion protection index of 98.6%, compared with only 82% for the uncoated material. Antibacterial tests showed that the TiO_2_-ZnO material suppressed the growth of populations of the organisms *C. albicans* (1.5·× 10^5^) and *E. coli* (1.5·× 10^7^). In an experiment involving the photodegradation of methylene blue (C.I. Basic Blue 9), the TiO_2_-ZnO oxide system produced a high yield of dye decomposition (70% after exposure to UV light for 6 h).

Chamanzadeh et al. [164] described the synthesis of a TiO_2_-ZnO oxide system with potential applications in DSSCs. Similar properties of a TiO_2_-ZnO binary oxide material were reported by Shanmugam et al. [152]. A DSSC was built using the commercial dye N719 (di-tetrabutylammonium cis-bis(isothiocyanato) bis(2,2′-bipyridyl-4,4′-dicarboxylato)ruthenium(II)). Based on obtained electrochemical curves it was shown that the ZnO layer improves adsorption of the dye and reduces the electron recombination rate. It was also found that the materials offered high photovoltaic efficiency (PCE = 8.3%) and charge accumulation at a level of 99% (for the material TiO_2_-(1)ZnO).

The combination of several catalytic techniques can provide greater possibilities of removing dangerous contaminants. The observed improvement in catalytic activity may be linked to a synergy effect, as has been widely described in literature [183,184,185]. The combination of techniques such as photocatalysis and sonocatalysis can significantly improve the efficiency of degradation of organic impurities using TiO_2_ [186,187]. Fatimah and Novirasari [165] described a simple synthesis of a binary photoactive material based on titanium dioxide. A combination of different catalytic techniques was studied with the aim of improving the catalytic properties. The material obtained was found to exhibit high photoactivity. A synergy effect was observed between photocatalytic degradation (yield 47%) and sonocatalytic degradation (yield 37%): the yield of phenol degradation in a sonophotocatalytic process was as high as 99%. 

The following part of this review will discuss binary oxide systems based on TiO_2_ and ZrO_2_, again synthesized using the sol-gel method. A summary is given in Table 5.

The use of polymer matrices such as the commercial Pluronic P123 or F127 (poly(ethylene glycol)-block-poly(propylene glycol)-block-poly(ethylene glycol)—PEG-PPG-PEG) is becoming more and more popular in the synthesis of titania-based materials [169,170,171,172,173,174,175,176,177,178,179,180,181,182,183,184,185,186,187,188,189,190,191,192,193,194,195,196,197,198]. Fan et al. [29] developed a method of synthesizing a TiO_2_-ZrO_2_ oxide system using Pluronic123 (P123) and Macrogol20000 (PEG) matrices. In the first stage, Pluronic P123 and Macrogol20000 were added to titanium tetrabutoxide. Then a zirconium precursor was added to the mixture, to achieve the molar ratio Ti:Zr = 1:0.1. The product underwent aging for 24 h at room temperature, and was then dried and calcined at 800 °C for 5 h. Diffractograms showed characteristic peaks for anatase, rutile and tetragonal zirconium dioxide. BET surface areas of 136.9, 138.5 and 148.9 m^2^/g were measured for the systems TiO_2_-ZrO_2_(P123 + PEG), TiO_2_-ZrO_2_(PEG) and TiO_2_-ZrO_2_(P123) respectively. The synthesized oxide materials were tested in the photodegradation of rhodamine B (C.I. Basic Violet 10) in UV light and in darkness. The materials demonstrated high photocatalytic efficiency: 91.93%, 91% and 90% for TiO_2_-ZrO_2_(P123 + PEG), TiO_2_-ZrO_2_(PEG) and TiO_2_-ZrO_2_(P123) respectively. 

Environmental pollution by harmful organic compounds is a severe problem for both humans and animals. Acetone is one of the most serious air pollutants in indoor environments, and its catalytic decomposition to less harmful compounds is a problem of interest to many research centres worldwide. Hernandez-Alonso et al. [188] obtained a TiO_2_-ZrO_2_ oxide system in the form of thin films, which they tested for photocatalytic activity in the degradation of acetone. Two methods of synthesis were investigated: sample M1, in which Ti_0.9_Zr_0.1_O_2_ was obtained by simultaneous hydrolysis of titanium tetraisopropoxide and zirconium tetrapropoxide; and sample M2, in which Ti_0.9_Zr_0.1_O_2_ was produced by polymerization of metal alkoxides in n-propanol. Thin layers of the binary oxide materials may be used successfully to coat Raschig rings, for example, and applied in various catalytic processes. The results of photo-oxidation of acetone confirmed the good photocatalytic properties of the synthesized materials. The method of synthesis (M1 or M2) was not found to affect the photocatalytic activity. 

**Figure 10:**
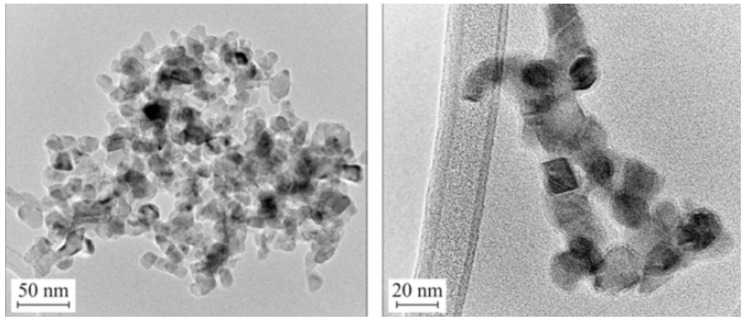
HRTEM images of the oxide system TiZr6 calcined at 700 °C (created based on [189] with permission from Springer).

Kraleva et al. [189] described a simple method of obtaining a TiO_2_-ZrO_2_ oxide system using metal alkoxides (titanium tetraisopropoxide and zirconium tetrapropoxide). In the first stage the TiO_2_ and ZrO_2_ precursors underwent hydrolysis in a solution of propan-2-ol and ammonia, and the resulting sols were subjected to aging for 24 h. The TiO_2_-ZrO_2_ system was formed by mixing the sols, with the molar contribution of zirconium dioxide equal to 3, 6, 13 and 37 (the samples were denoted TiZr*n*, where *n* was the molar contribution of ZrO_2_). The resulting systems were dried at 100 °C for 24 h, and calcined at 550 and 700 °C for 5 h. The final binary oxide materials had a similar crystal structure to those described in earlier work. X-ray diffractograms contained characteristic peaks for anatase and rutile. For the system TiZr37 calcined at 700 °C, the presence of srilankite (Ti_0.63_Zr_0.37_O_x_) was also bserved. The calcination temperature was found to have a significant effect on the crystaline structure, with anatase-rutile transformation at 700 °C. TEM images (Figure 10) showed particles with a spherical shape, having a marked tendency to agglomerate. 

The BET surface areas of the TiO_2_-ZrO_2_ systems calcined at different temperatures were 26, 37, 40 and 172 m^2^/g for 550 °C and 26, 29, 30 and 36 m^2^/g for 700 °C, respectively for the systems denoted TiZr3, TiZr6, TiZr13 and TiZr37. The calcination temperature was found to affect the porous structure parameters: with an increase in temperature, the BET surface area decreased (this is related to the effect of sintering). 

Atmospheric pollution is one of the most urgent present-day problems. A priority task in the synthesis of new nanomaterials is the possibility of controlling exhaust emissions. Titania-based gas sensors for the quantitative detection of various toxic and harmful gases have been widely developed in view of their high response, outstanding selectivity, excellent repeatability, and good stability. Mohammadi and Fray [190] described the use of a system combining titanium dioxide and zirconium dioxide in detecting nitrogen(IV) oxide and carbon(II) oxide. Results obtained for the sensoric properties of the materials showed that the highest response to the applied concentrations of NO_2_ (2 ppm) and CO (100 ppm) was given by the system that had been calcined at 500 °C (the response was 9.1 for CO and 5.1 for NO_2_ at a temperature of 150 °C). Detailed analysis of the morphological and crystal structures was carried out using SEM and XRD. In the crystalline structure analysis, diffraction peaks for anatase and zirconium dioxide were obtained. Based on the X-ray analysis, the average crystallite size was computed to be 5.8 nm (500 °C) and 8.5 nm (900 °C). The SEM images showed particles with a spherical shape, having a marked tendency to agglomerate. The average particle size was 20 nm (500 °C) and 36 nm (900 °C).

Naumenko et al. [191] described the synthesis of a TiO_2_-ZrO_2_ oxide system using a sol-gel method, with titanium tetraisopropoxide and zirconium tetrapropoxide as precursors of the oxides. The first stage of the synthesis was the hydrolysis of Ti[OCH(CH_3_)_2_]_4_ and Zr[OCH(CH_3_)_2_]_4_ in a mixture of deionized water and hydrochloric acid. The prepared sols were then mixed, with the Ti[OCH(CH_3_)_2_]_4_:Zr[OCH(CH_3_)_2_]_4_ molar ratio ranging from 1:0 to 0.7:0.3. The resulting materials underwent aging for 12 h, and were then calcined at 400 and 500 °C in air. The products were found to contain only the crystal structure of anatase. Detailed analysis of the results obtained for crystal structure parameters showed a change in the parameters when the molar fraction of ZrO_2_ was 0.3, with values of a = 3.82 Å, c = 9.726 Å (ASTM 84-1286: a = 3.7822 Å, c = 9.5023 Å). This may indicate that the ZrO_2_ was incorporated into the TiO_2_ structure to form Ti_1−x_Zr_x_O_2_. AFM images showed the TiO_2_ particles to have diameters of 10–15 nm. An increase in the molar fraction of ZrO_2_ led to the appearance of particles with diameter 20 nm.

It is well known that in order to obtain a binary oxide material based on titania with strictly defined properties, it is necessary to choose appropriate synthesis conditions. Parameters such as the molar ratio or the temperature of thermal treatment can have a decisive influence on the resulting material. Kokporka et al. [27] investigated the effect of the molar ratio of the oxides and the temperature of calcination on the physico-chemical properties of a TiO_2_-ZrO_2_ system. They also determined the photocatalytic activity of the materials in the evolution of hydrogen. Diffractograms showed the presence of characteristic peaks for the crystal structures of anatase and rutile. The crystal structure parameters were found to be affected by the temperature of thermal treatment and by the molar ratio. Scanning electron microscope images showed particles of spherical shape with a marked tendency to agglomerate. The BET surface area, determined by low-temperature nitrogen sorption, was 130 m^2^/g for the systems 0.95TiO_2_-0.05ZrO_2_ and 0.2TiO_2_-0.8ZrO_2_, and 190 m^2^/g for 0.4TiO_2_-0.6ZrO_2_ and 0.6TiO_2_-0.4ZrO_2_. In the tests of photocatalytic activity, high yields of hydrogen were obtained in the presence of TiO_2_-ZrO_2_. The highest yield (0.61 mL/h·g) was obtained using the oxide system 0.95TiO_2_-0.05ZrO_2_ calcined at 800 °C.

Due to their high chemical and thermal stability, both Titania [199] and Zirconia [200] are used as matrices for the application of nanoparticles. Karthika et al. [192] described an application of a TiO_2_-ZrO_2_ binary material as a matrix for Sm^3+^ and CdS nanoparticles. TEM images showed a crystallite size of 7.8 nm and an interplanar distance of 3.533 Å, which corresponds to the CdS (100) plane. FTIR spectra contained bands for Ti–O–Ti at the wavenumber 440 cm^−1^, Zr–O–Ti at 663 cm^−1^ and Zr–O–Zr at 841 cm^−1^. Optical absorption and emission spectra confirmed the presence of CdS nanoparticles along with Sm^3+^ ions in the TiO_2_-ZrO_2_ matrices.

**Figure 11:**
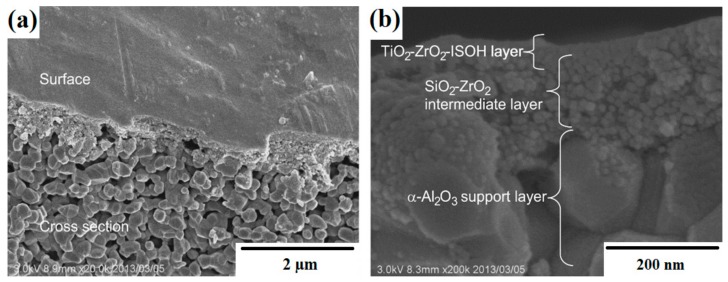
SEM images of surface and cross-section views of a TiO_2_–ZrO_2_–ISOH membrane calcined at 350 °C under N_2_: (**a**) surface and cross-section views at 20,000×; (**b**) cross-section view at 200,000× (created based on [193] with permission from Elsevier Publisher).

Due to the ever faster development of industry, it is necessary to obtain effective membranes for gas separation processes, such as the separation of CO_2_ and purification of organic gases under normal temperature and pressure conditions [201]. Therefore, interest in inexpensive inorganic materials such as Titania [202] and zirconia [201,203] is increasing. Fukumato et al. [193] described the use of the chelating complexes DEA and isoeugenol (ISOH) in the synthesis of a TiO_2_-ZrO_2_ oxide system. The goal of the study was to obtain amorphous microporous membranes. The single-stage synthesis involved the hydrolysis of titanium tetraisopropoxide and zirconium tetrapropoxide in a water solution of propan-1-ol. Two forms of material were obtained: with the addition of diethylamine (TiO_2_-ZrO_2_-DEA) and with the addition of isoeugenol (TiO_2_-ZrO_2_-ISOH). The synthesis product was used to prepare a membrane on a base made from Al_2_O_3_ and SiO_2_-ZrO_2_. The final stage of the process was calcination of the TiO_2_-ZrO_2_ system in membrane form at 500 and 600 °C in air and at 350 and 400 °C in a nitrogen atmosphere. The TiO_2_-ZrO_2_ membranes were tested for permeability to the gases He, N_2_, CO_2_ and N_2_. The oxide systems calcined in air exhibited higher permeability than those calcined in nitrogen. This is probably due to residues of the chelating compounds present in the nitrogen-calcined systems. The permeabilities of the TiO_2_-ZrO_2_-ISOH membranes were higher than those of the TiO_2_-ZrO_2_-DEA membranes, and were equal to 110, 48 and 50 [10^−7^ mol/m^2^·s·Pa] for He, CO_2_ and N_2_ respectively. Figure 11 shows SEM images of the membrane.

Anodized aluminium oxide (AAO), due to properties such as its nano-sized, self-organized, hexagonal porous structure [204], is an interesting material as a substrate for titania-based materials. Such materials can be used, for example, as sensors for hydrogen detection [204,205,206]. Qu et al. [26] investigated the use of anodic aluminium oxide as a matrix to obtain a photoactive TiO_2_-ZrO_2_ oxide system. In the first stage, AAO was immersed in zirconium nitrate for 12 h at 80 °C, after which it was dried for 1 h at 80 °C. The ZrO_2_-grafted AAO was then immersed in TiF_4_ for 9 min at 60 °C. In the final stage, the product was calcined in air for 3 h at 600 °C. Diffractograms showed the presence of characteristic diffraction peaks for anatase and for monoclinic zirconium dioxide. SEM images showed particles in the form of densely packed nanotubes with a tendency to agglomerate. Analysis of porous structure parameters showed the BET surface area of the oxide system to be 47.4 m^2^/g. The band gap of the system was measured at 3.22 eV. The synthesized material was tested as a catalyst in the photodegradation of methyl orange (C.I. Basic Orange 10). A high yield of photodegradation of the dye was obtained, amounting to 96% after 180 min, compared with 83% for TiO_2_ and 45% for ZrO_2_.

Another team of researchers [194] described the use of a TiO_2_-ZrO_2_ oxide system to obtained hydroxymethylfufural (HMF) from biomass. HMF is a precursor of materials such as polyesters and polymers based on furfural, as well as diesel fuels [207,208]. The binary oxide system was obtained in a single-stage sol-gel process, with the simultaneous hydrolysis of titanium tetrabutoxide and zirconium tetrabutoxide. The material was then dried at 120 °C for 12 h and calcined at 500 °C for 12 h. Diffractograms showed the presence of peaks corresponding to the structures of anatase and of monoclinic and tetragonal zirconium dioxide. When the molar fraction of ZrO_2_ was increased, the anatase structure became less visible. Measurements of the porous structure parameters gave BET surface areas of 68, 62 and 60 m^2^/g and pore volumes of 0.27, 0.18 and 0.13 cm^3^/g respectively for the systems TiO_2_-ZrO_2_(3/1), TiO_2_-ZrO_2_(1/1) and TiO_2_-ZrO_2_(1/3). Tests of glucose conversion gave yields of 72–86%, the main product being HMF; its maximum yield was obtained using the system TiO_2_-ZrO_2_(1/1). The use of tetrahydrofuran (THF) as a co-solvent and NaCl as the aqueous phase caused a tripling of the HMF conversion yield. The HMF yield varied depending on the co-catalysts: Amberlyst 70 (85.6%) > Nafion NR50 (73.4%) > Cs_3.5_SiW (35.2%) > Cs_2.5_PW (31.8%). 

One of the pollutants of greatest importance in drinking water treatment is humic acid [209]. Many attempts have been made to use low-pressure driven membrane systems for the removal of the largest fractions of humic acid. The recently widely described photocatalytic membrane reactor is an interesting alternative to conventional techniques [210,211,212]. Khan et al. [195] described the synthesis of a TiO_2_-ZrO_2_ binary oxide system as an active photocatalyst for removing humic acid in membrane reactors. The TiO_2_-ZrO_2_ system achieved 85% efficiency in the removal of total organic carbon. The effect of double-positive ions (Ca^2+^) on the efficiency of the process was also investigated: addition of these ions caused an increase in the efficiency of removal of total organic carbon, possibly due to the reduced electrostatic repulsion between humic acid and the TiO_2_-ZrO_2_ system. The last part of the study concerned the effect of UV radiation, which has oxidizing properties, on a PVDF membrane placed in a reactor. FTIR spectra demonstrated that the structure of the PVDF membrane did not undergo an oxidation process under irradiation with UV light.

Literature reports on the synthesis of TiO_2_-ZnO and TiO_2_-ZrO_2_ oxide systems by sol-gel routes confirm that this technique provides the possibility of controlling the process through appropriate selection of process conditions, and thus enables control of the physico-chemical properties of the final product (well-defined particle size, crystal structure and surface activity). A suitable choice of process conditions (applied reagents such as organic and inorganic precursors, solvent type, type of hydrolysing and/or nucleating agents, molar ratio of oxides, pH of the reaction system, rate and direction of reagent dosing, time and temperature of the process, conditions of final heat treatment, etc.) can lead to oxide materials based on titanium dioxide together with zinc oxide or zirconium dioxide with precisely designed structure and texture. This has a significant impact on the possible applications of the produced systems in such fields as photocatalysis, electrochemistry and adsorption. 

### 2.3. The Electrospinning Method 

**Figure 12:**
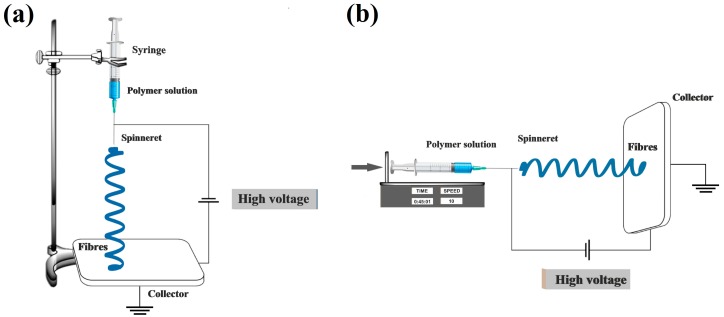
Schematic diagrams of (**a**) vertical and (**b**) horizontal electrospinning processes (created based on [219] with permission from Elsevier Publisher).

Another developing method of synthesis of oxide systems containing titanium dioxide is the electrospinning method, which represents a continuation of research into electrosprays. The first work on an electrospray was reported by Rayleigh, who observed that a droplet subjected to high voltage disintegrates due to the potential gradient. This line of research was taken up by Zeylen and Dole [213,214], who described droplets dissociated in an electric field to form an aerosol. The physical principles behind the two processes are similar, the only difference being the form of the product obtained: nanofibers in the case of electrospinning, and nanodroplets in the case of electrosprays. The apparatus used to obtain nanofibers by electrospinning consists of a high voltage source in the range 1–30 kV, a solution feeder (a syringe and needle), a pump and a collector [215,216,217,218]. Schematic diagrams of vertical and horizontal electrospinning processes appear in Figure 12.

In electrospinning, a solution is placed in a syringe, and then a high voltage is applied to the needle or nozzle, producing electrostatic forces which cause movement of the charges in the solution towards the collector. A dosing pump, as well as exceeding of the critical value of the field intensity, initiates the outward flow of the solution towards the collector. Under the influence of the applied voltage, the liquid is stretched into a thin stream. As the stream falls, the solvent evaporates, and the resulting nanofibers are accumulated on the collector [220,221,222]. 

As in the case of the other methods that we have described, such as the hydrothermal and sol-gel methods, the parameters of the electrospinning process may affect the physico-chemical properties of the final product [223,224,225,226]. Table 6 illustrates the process parameters impacting the result of electrospinning, relating to the solution, the apparatus and the external environment. 

The chief advantages of the electrospinning method include the relatively simple apparatus and the fast production time. Another advantage of this method is the ability to produce nanofibers, for both laboratory and industrial use. The greatest drawbacks, however, are the instability of the stream, which affects the diameter of the resulting nanofibers, the fact that individual fibers may stick together, and the need for the polymer used in electrospinning to be soluble in the reaction medium [227,228,229,230,231].

Because of its advantages, electrospinning is becoming an interesting alternative method for obtaining oxide systems containing titanium dioxide. There has recently been a marked increase in interest in the use of electrospinning to produce oxide systems such as TiO_2_-ZnO and TiO_2_-ZrO_2_, or TiO_2_-MoS_2_ hybrid systems. 

In the next part of this review, a survey will be made of work in which TiO_2_-ZnO oxide systems were obtained by the electrospinning method (Table 7).

Ammonia is a useful chemical material, widely used in many industries. However, it is a common material in industrial effluents and very dangerous to the environment and humans [239,240]. It attacks the respiratory system, skin, and eyes, and at a concentration higher than 300 ppm may lead to death [241,242]. Therefore, it is necessary to develop new materials to detect this type of impurity. Nanofibers possess favorable properties such as small diameters and large specific surface areas. For gas sensors, surface area is one of the most important factors influencing sensing performance [243]. Wang et al. [232] described the use of the electrospinning method for the saturation of a binary TiO_2_-ZnO material with sensory properties. The first stage was the preparation of a solution of precursors of the oxides and acetic acid with the addition of poly(vinylpyrrolidone). The solution was then transferred to a syringe pump and a voltage of +7 kV was applied. The spinning rate was 0.3 cm^3^/h. The oxide material was calcined at 500 °C for 3 h, and then immersed in a 0.1 mol/L solution of FeCl_3_ for 30 min, dried, and subjected to the action of polypyrrole (PPy) for 5 h, to produce the oxide system TiO_2_-ZnO-PPy. Analysis of XPS spectra showed the presence of Ti2p, O1s and Zn2p bands. SEM images of the PPy-modified materials showed the oxide system to have the form of black nanofibers with diameter 180 nm and lengths of several millimeters. The unmodified fibers had a uniform smooth surface. TEM images showed the oxide material to have a core-shell structure, where the thickness of the PPy shell was 7 nm. The TiO_2_-ZnO-PPy system was tested as an ammonia sensor. It was found to have a fast response rate and a detection limit of 60 ppb. The results obtained were probably influenced by the reduction of diffusion resistance caused by the porous PPy shell.

Park et al. [233] described a combination of the methods of electrospinning and atomic layer deposition (ALD) in the synthesis of a TiO_2_-ZnO binary oxide system. In the first stage, TiO_2_ nanofibers were obtained by electrospinning from a solution of titanium tetraisopropoxide and poly(vinyl acetate), with an applied voltage of +10 kV and a constant spinning rate of 0.2 cm^3^/h. The resulting nanofibers were calcined at 600 °C for 8 h. The TiO_2_ fibers were then coated with ZnO using the ALD method. The number of ALD cycles was in the range 50–400. Diffractograms showed the TiO_2_-ZnO system to contain the crystal structure of anatase and wurtzite. The HRTEM results were consistent with those of XRD. SEM images showed that the increment in the ZnO layer per ALD cycle was 0.66 nm. Based on EDS (Energy-dispersive X-ray spectroscopy) mapping a core-shell structure was demonstrated, with a core of TiO_2_ and a shell of ZnO. As in the work of Wang et al. [232], the obtained TiO_2_-ZnO nanofibers were tested as a chemical sensor. Park et al. [233] assessed the sensory properties of their product using oxygen. It was found to have good properties for the detection of oxygen, and the sensor’s response varied with changes in the oxygen concentration. The tested chemical sensor demonstrated high stability and repeatability.

Surveys of the subject literature have already shown that binary oxide materials such as TiO_2_-ZnO obtained by the hydrothermal method [62] and by the sol-gel method [156,161,163] have good photocatalytic properties in the degradation of methylene blue (C.I. Basic Blue 9). Materials of the same type synthesized by other methods can be expected to exhibit similar properties. A team of researchers [234] described the synthesis of a TiO_2_-ZnO system by electrospinning and its use to catalyze the photodegradation of C.I. Basic Blue 9. X-ray diffraction results showed the presence of reflection corresponding to the structure of anatase, wurtzite and zinc titanate. From TEM images, the mean particle diameter was determined to be 20 nm. Using low-temperature nitrogen sorption, the BET surface area was measured at 203 m^2^/g and the pore volume at 0.24 cm^3^/g. The TiO_2_-ZnO oxide system synthesized by electrospinning demonstrated high photocatalytic activity, at a similar level to the same material produced using other methods.

Rhodamine B is widely used as a colorant in textiles and foodstuffs, and is also a well-known water tracer fluorescent [244]. Many scientific studies have demonstrated the harmful effects of rhodamine B on humans and the environment. Its carcinogenicity, reproductive and developmental toxicity, neurotoxicity and chronic toxicity to humans and animals have been experimentally proven [245,246,247]. Due to the harmful effects of this material, it is necessary to develop new nanomaterials enabling its effective degradation [248,249]. Liu et al. [235] used cellulose acetate as a matrix for the synthesis of TiO_2_-ZnO oxide systems with TiO_2_:ZnO mass ratios of 87.6:12.4, 84.2:15.8 and 77.9:22.1. XRD results showed the presence of diffraction peaks corresponding to anatase, wurtzite and zinc titanate. Based on TEM images, the interplanar distance was measured at 3.56 Å, corresponding to dimension *d* of the (101) plane of the anatase crystal structure. XPS spectra showed the presence of Ti2p, Zn2p and O1s bands. The obtained materials were used in the process of photodegradation of rhodamine B (C.I. Basic Violet 10) and phenol. All of the oxide systems demonstrated higher photocatalytic activity than pure TiO_2_. The highest photodegradation yield was obtained in the presence of the material synthesized in the molar ratio TiO_2_:ZnO = 84.2:15.8. The researchers suggested that excessive ZnO may restrict access to recombination sites, thus reducing the photocatalytic performance. 

Kanjwal et al. [236] compared TiO_2_-ZnO binary oxide materials synthesized by the hydrothermal and electrospinning methods. In the electrospinning process, vinyl acetate was dissolved in DMF, and then titanium tetraisopropoxide, zinc oxide and acetic acid were added. The solution was transferred to a syringe, and a voltage of +12 kV was applied. The material was dried at 80 °C for 24 h and calcined at 600 °C for 1 h. Hydrothermal synthesis was carried out at 150 °C for 1 h, using nanofibers of TiO_2_ and zinc nitrate. Diffractograms revealed the characteristic crystal structures of wurtzite (after electrospinning) and anatase (after hydrothermal synthesis). SEM and TEM images showed that the electrospun oxide system consisted of nanofibers with a smooth surface, while in the case of the material obtained hydrothermally, ZnO nanoparticles were observed on the TiO_2_ surface. Physico-chemical analysis of the binary oxide materials showed marked differences in their morphological and crystal structure. Their photocatalytic properties were assessed in the degradation of rhodamine B (C.I. Basic Violet 10). Both the electrospun and hydrothermally synthesized materials demonstrated superior photocatalytic activity to pure TiO_2_ or ZnO. The hydrothermally treated materials produced higher degradation yields (100% after 105 min of exposure to light) than the electrospun materials; this may be linked to differences in the crystal structures of the oxide systems.

Today, various chemical pollutants such as dyes are attracting increased attention owing to their significant effects on public health and the environment. The efficient degradation of this type of pollution from wastewater is becoming an urgent and challenging problem. Many centers are conducting research on the use of electrospinning for the synthesis of titania-based materials and their use in wastewater photodegradation [250,251,252]. Li et al. [237] developed a synthesis process based on electrospinning for a photocatalytic TiO_2_-ZnO oxide system. The resulting system demonstrated very good photocatalytic properties in the degradation of rhodamine B (C.I. Basic Violet 10), producing a higher degradation yield than pure TiO_2_ or ZnO. It also exhibited high photocatalytic stability over five consecutive cycles. More detailed physico-chemical analysis was performed using the XRD, SEM and TEM techniques. X-ray diffraction results demonstrated the presence of characteristic diffraction peaks corresponding to anatase and wurtzite. The SEM and TEM images showed smooth nanofibers consisting of single nanoparticles. 

**Figure 13:**
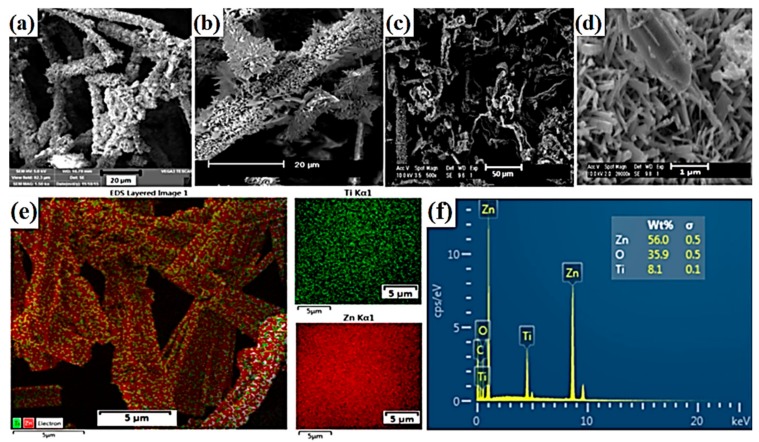
Images of TiO_2_-ZnO oxide materials: (**a**,**b**) SEM for samples before calcination; (**c**,**d**) SEM for calcined materials; (**e**) EDS mapping and (**f**) EDX spectrum (created based on [238] with permission from Springer).

Araújo et al. [238] synthesized a TiO_2_-ZnO oxide material using the methods of electrospinning and atomic layer deposition. In the first stage, a solution of TiO_2_ (P25) and poly(methacrylic acid-co-methyl methacrylate) 1:1 block copolymer in ethanol was subjected to a constant voltage of +15 kV for 5 min. Next, zinc acetate was dissolved in water and mixed for 15 min at 85 °C, after which triethylamine was added and the mixture incubated for 3 h (solution 1). At the same time, zinc acetate was dissolved in deionized water with the addition of hexamethyltetramine (solution 2). The obtained TiO_2_ nanofibers were immersed in solution 1, dried at 120 °C for 1 h, then added to solution 2 and kept at 85 °C for 24 h. The synthesized oxide material was calcined for one hour at 500 °C. Diffractograms revealed the presence of characteristic reflections for the crystalline structure of anatase, rutile and wurtzite. SEM images (Figure 13a–d) showed that the TiO_2_ nanofibers were coated with ZnO nanorods. The EDX spectrum (Figure 13e–f) showed characteristic bands for Ti, O and Zn. The materials were tested as a sensor for the detection of moisture. Materials with one and three layers of ZnO produced a high response to changes in humidity. The three-layer material exhibited significantly greater sensitivity in the humidity range 40–100%. It also demonstrated significantly better parameters in moisture detection than the sensors described in a previous study [253]. The researchers suggest that the increase in ZnO content improves the porous structure, and thus increases the sensitivity [253].

Table 8 contains a review of the literature concerning the synthesis of TiO_2_-ZrO_2_ oxide systems using the electrospinning method.

**Figure 14:**
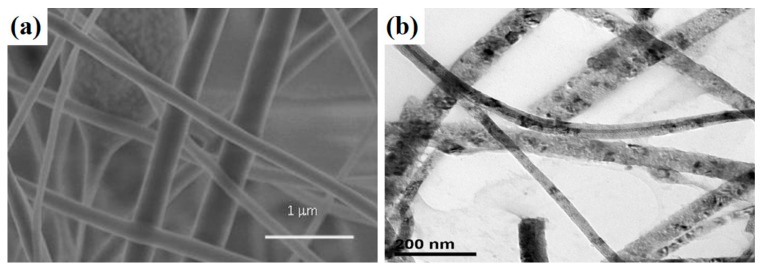
(**a**) SEM image and (**b**) TEM image of the TiO_2_-ZrO_2_ oxide system (created based on [254] with permission from Elsevier Publisher).

Humidity is a very common component in our environment that can affect not only human life, but also many industrial processes. Therefore, the measurement and control of humidity are of great importance. The design of a successful humidity sensor is a complex process, as the material used to build the sensor must offer properties including linear response, high sensitivity, fast response time, chemical and physical stability, wide operating range and low cost. Su et al. [254] developed a simple synthesis method involving electrospinning of a TiO_2_-ZrO_2_ oxide system with defined moisture detection properties. The impedance was shown to differ by four orders of magnitude, from 10^5^ to 10^1^ kΩ, in the humidity range 11–97%, which indicates correct operation of the sensor. Good repeatability was demonstrated for the obtained results, and the influence of temperature on the sensor was shown to be small. To determine the physico-chemical characteristics of the sensor material, analysis was performed using XRD, SEM and TEM. The XRD results indicated the crystal structure of anatase and tetragonal zirconium dioxide. Based on the SEM images it was concluded that the binary oxide material contained fibers originating from both ZrO_2_ and TiO_2_. The diameters of the nanofibers were found to be in the range 240–400 nm in the case of ZrO_2_, and 60–200 nm in the case of TiO_2_. Transmission electron microscopy results (Figure 14) showed the nanofibers to be built of numerous small particles. 

Recent years have seen a marked increase in interest in proton exchange membrane fuel cells (PEMFCs). The development of the ionomer membrane is crucial for the reliability of such cells and their high-volume commercialization. This type of membrane must attain a high proton conductivity and a low gas permeability, offering at the same time high mechanical, chemical and thermal stability, a low degree of deformation due to water absorption, and low costs of production so that it can be used globally [256,257,258]. Lee et al. [255] described the use of a TiO_2_-ZrO_2_ binary oxide system to prepare aquivion (KNF Neuberger GmbH) membrane for use in a single fuel cell. In the course of comprehensive physico-chemical analysis, diffractograms were obtained which indicated an amorphous structure for the material calcined at 500 °C, and the crystal structure of srilankite (Ti_0.5_Zr_0.5_O_2_) for the oxide systems calcined at 700 and 1000 °C. In view of these results, further analysis was carried out only on the material with an amorphous structure, since that material had the highest specific surface area among the synthesized oxide systems. Based on SEM images, the average fiber diameter was measured at 497 nm before calcination and 344 nm after calcination. The synthesized TiO_2_-ZrO_2_ oxide system calcined at 500 °C was modified with 85% phosphoric acid to increase its proton conductivity. The XPS spectrum before modification showed two bands at 530.35 and 532.8 eV, corresponding to O–M bonds (M = Ti, Zr) and O–H bonds. On the spectrum of the modified material a new band appeared at 531.36 eV, originating from P–O bonds. The use of the oxide material to build the membrane eliminated the effect of cracking, and the membrane modified with phosphate groups showed improved proton conductivity compared with pure TiO_2_-ZrO_2_ and aquivion. The composite membrane demonstrated good durability, as confirmed by the results of accelerated lifetime (ALT) measurements.

The above review of the literature on the formation of TiO_2_-ZnO and TiO_2_-ZrO_2_ oxide systems by the electrospinning method confirms that this is a new and promising method which leads to the effective synthesis of materials in the form of nanofibers with defined chemical composition, porosity, geometry and fiber dimensions. Moreover, the method does not place excessive limitations on researchers. The sole problem is the selection of optimum process conditions, including the type and concentration of solvent, temperature, solution viscosity, average molecular weight of polymer, applied voltage, and flow rate. It has been shown that electrospun TiO_2_-ZnO and TiO_2_-ZrO_2_ fibers may be used, among other things, as sensors for the detection of various types of gas, as photocatalysts in the degradation of certain types of organic pollutants, and as membranes for the production of fuel cells.

## 3. Conclusions

The literature review that has been presented here reflects the search for innovative combinations of TiO_2_ with other materials, which may cause morphological changes in the crystalline phases of titanium dioxide and changes in its electron structure, leading to—among other things—improvement in its photocatalytic activity and spectral sensitivity. Studies being carried out at multiple research centers worldwide are largely focused on the choice of appropriate methods for the synthesis or modification of titanium dioxide, and on the selection of suitable components to be combined with TiO_2_ to produce advanced multifunctional hybrid materials. 

It is widely known that the physico-chemical as well as the morphological-structural properties of materials based on titanium dioxide, such as particle shape and size, degree of crystallinity, surface composition, pore size distribution, specific surface area, etc., are strongly dependent on the suitable specification of synthesis conditions, such as the nature and composition of the precursors, solvent and complexing/templating agent, and the conditions of hydrolysis and calcination. Suitably chosen process conditions for the synthesis of oxide and hybrid materials based on TiO_2_ make it possible to design their physico-chemical properties. 

Issues relating to the creation of a new generation of modified forms of TiO_2_ are of huge importance, particularly in view of the growing need for active photocatalysts functioning in visible light, as well as high-performance electrode materials.

## Figures and Tables

**Table 1:** Resources, synthesis conditions and physico-chemical properties of TiO_2_-ZnO oxide systems obtained by the hydrothermal method.

Resources	Synthesis Conditions	Physico-Chemical Properties of the Final Product	References
Ti(OC_4_H_9_)_4_, Zn(CH_3_COO)_2_·2H_2_O, ethanol, deionized water	hydrothermal treatment: 120, 180, 200 °C, reaction time: 1, 12, 24, 48 h, drying: 60 °C, calcination: 350 °C for 1 h	anatase crystal structure, spherical shaped particles with a tendency to agglomerate and particle sizes in the range 413–527 nm	[8]
TiO_2_ microspheres (P25 Degussa), Zn(NO_3_)_2_·6H_2_O, hexamethylenetetramine, distilled water	hydrothermal treatment: 100 °C, reaction time: 1–5 h, drying: 60 °C	crystal structure of anatase and wurtzite, spherical shaped particles coated with nanospindles, particle diameter 1.5–2 μm, BET surface area 122.2–34.8 m^2^/g (reduction of specific surface area with increase of ZnO fraction)	[56,57]
Ti(SO_4_)_2_, ZnCl_2_, NH_3_·H_2_O, cetyltrimethylammonium bromide, distilled water	hydrothermal treatment: 100 °C, reaction time: 4 h, drying: 60 °C	crystal structure of TiO, Ti_3_O_5_ and ZnO, particles with a smooth surface with a tendency to agglomerate	[58]
Ti[OCH(CH_3_)_2_]_4_, Zn(NO_3_)_2_·6H_2_O, NaOH, distilled water, poly(vinyl alcohol)	hydrothermal treatment: 220 °C, reaction time: 5 h, drying: 80 °C for 2 h	crystal structure of anatase and wurtzite, particles with spherical and cubic shapes with a tendency to agglomerate, particle diameter 30 nm	[59]
Ti(OC_4_H_9_)_4_, Zn(CH_3_COO)_2_·2H_2_O, acetic acid, ethylene glycol, deionized water	hydrothermal treatment: 180, 190, 200 °C, reaction time: 8, 12, 15, 24 h, drying: 60 °C	crystal structure of anatase and wurtzite, particle size distribution in the range 25–100 nm with average particle diameter 64 nm, BET surface area 97 m^2^/g and 206 m^2^/g for systems obtained at 180 and 200 °C respectively	[60]
Ti(SO_4_)_2_, Zn(NO_3_)_2_·6H_2_O, NH_4_F, CO(NH_2_)_2_, ethanol, distilled water	hydrothermal treatment: 120-180 °C, reaction time: 2–24 h drying: 80 °C for 24 h	crystal structure anatase and wurtzite, spherical particles with a tendency to agglomerate, nanoparticles of diameter 0.5–2 µm	[61]
Ti[OCH(CH_3_)_2_]_4_, Zn(CH_3_COO)_2_·2H_2_O, NH_3_·H_2_O, ethylene glycol	hydrothermal treatment: 220 °C, reaction time: 5 h, drying: 60 °C	crystal structure of anatase and wurtzite, particles with a tendency to agglomerate, porous microstructure	[62]
TiCl_4_, ZnCl_2_, urea, ethanol, distilled water	hydrothermal treatment: 180 °C, reaction time: 16 h, calcination: 450 °C for 2 h	crystal structure of anatase and wurtzite, particles taking the shape of nanorods with a tendency to agglomerate	[63]
TiCl_4_, Zn(NO_3_)_2_·6H_2_O, NH_3*_H_2_O, ethanol, distilled water	hydrothermal treatment: 150 °C, reaction time: 1 h, freeze-drying: −55 °C in vacuum, calcination: 600, 700, 900 °C for 1 h	crystal structure of rutile, wurtzite, Zn_2_TiO_3_O_8_, spherical particles with a tendency to agglomerate, particle diameters in the range 140–270 μm	[64]
Ti[OCH(CH_3_)_2_]_4_, Zn(CH_3_COO)_2_·2H_2_O, sodium hydroxide, deionized water	microwave treatment: 180 °C for 5 min, frequency: 2.45 GHz, calcination: 500, 600 °C	crystal structure of anatase, wurtzite and zinc titanates (ZnTiO_3_, Zn_2_Ti_3_O_4_), spherical and hexagonal particles with a tendency to agglomerate, mean particle size 36 and 31 nm for samples calcined at 500 and 600 °C	[65]
Ti(OC_4_H_9_)_4_, Zn(CH_3_COO)_2_, deionized water	microwave treatment: 80 °C for 30 min, drying: 80 °C	crystal structure of anatase and wurtzite, band gap energy 3.15 eV, spherical shaped particles with a tendency to agglomerate, BET surface area 290 m^2^/g, pore diameter 3.4 nm, pore volume 0.32 cm^3^/g	[66]

**Table 2:** Resources, synthesis conditions and physico-chemical properties of TiO_2_-ZrO_2_ oxide systems obtained by the hydrothermal method.

Resources	Synthesis Conditions	Physico-Chemical Properties of the Final Product	References
TiO_2_, ZrOCl_2_·8H_2_O, NaOH, distilled water	hydrothermal treatment: 180 °C, reaction time: 48 h, drying: 60 °C, calcination: 500 °C for 3 h	crystal structure of TiO_2_ and tetragonal ZrO_2_, nanorod particles up to 5 μm in length	[82]
Ti[OCH(CH_3_)_2_]_4_, Zr[OCH(CH_3_)_2_]_4_, propan-2-ol	hydrothermal treatment: 240 °C, reaction time: 24 h, drying: 100 °C for 2 h, calcination: 450 °C for 4 h	crystal structure of anatase, rutile and tetragonal ZrO_2_, spherical particles with a tendency to agglomerate	[83]
Ti[OCH(CH_3_)_2_]_4_, Zr[OCH(CH_3_)_2_]_4_, 1,3-butanediol, ethylene glycol	hydrothermal treatment: 300 °C, reaction time: 2 h, drying: 60 °C, calcination: 500 and 800 °C	crystal structure of tetragonal ZrO_2_, spherical particles with diameter up to 1 μm	[84]
TiOSO_4_, Zr(SO_4_)_2_, distilled water	hydrothermal treatment: 200, 240 °C, reaction time: 24 h, drying: 60 °C, calcination: 400–1000 °C for 1 h	anatase crystal structure (for systems obtained at a molar ratio of TiO_2_ > 50%) and monocrystalline ZrO_2_ (for all synthesized systems), spherical particles with diameter 13 nm	[85]
Ti[OCH(CH_3_)_2_]_4_, Zr[OCH(CH_3_)_2_]_4_, Zn(NO_3_)_2_·6H_2_O, propan-2-ol, nitric acid	hydrothermal treatment: 240 °C, reaction time: 24 h, drying: 100 °C for 2 h, calcination: 450 °C for 4 h	crystal structure of anatase, rutile and monolithic ZrO_2_, average crystallite size 17.4 nm, band gap for TiO_2_-ZrO_2_ = 2.15 eV	[86]
Ti[OCH(CH_3_)_2_]_4_, ZrOCl_2_·8H_2_O, poly(methyl methacrylate), ethanol	hydrothermal treatment: 160 °C, reaction time: 10 h, drying: 100 °C, calcination: 800 °C	crystal structure of anatase and monolithic ZrO_2_ (for a calcined system), particles with a structure of hollow microspheres, diameters in the range 0.7–2 μm, BET surface area 224.6 m^2^/g	[87]
TiCi_4_, ZrOCl_2_·8H_2_O, NH_3_·H_2_O, distilled water	hydrothermal treatment: 220 °C, reaction time: 4 h, drying: 120 °C, calcination: 500 °C for 10 h	amorphous structure, high thermal stability (10% mass loss), BET surface area 209 m^2^/g	[88]
commercial Ti, ZrOCl_2_·8H_2_O, NH_3_·H_2_O, H_2_O_2_, HNO_3_, lactic acid	hydrothermal treatment: 180 °C, reaction time: 12 h, drying: 50 °C for 12 h	crystal structure of anatase and ZrO_2_, spherical and spindle shaped particles	[89]
Ti[OCH(CH_3_)_2_]_4_, ZrCl_2_, HNO_3_, methanol	hydrothermal treatment: 260 °C, reaction time: 12 h, drying: 80 °C for 24 h	crystal structure of anatase, BET surface area 40.6 m^2^/g, pore volume 0.183 cm^3^/g, pore diameter 5.7 nm	[90]

**Table 3:** Resources, synthesis conditions and physico-chemical properties of TiO_2_-MoS_2_ hybrid systems obtained by the hydrothermal method.

Resources	Synthesis Conditions	Physico-Chemical Properties of the Final Product	References
TiCl_4_, Na_2_MoO_4_·2H_2_O, thioacetamide, deionized water	hydrothermal treatment: 240 °C, reaction time: 24 h, drying: 60 °C	crystal structure of TiO_2_ (anatase) and MoS_2_, layered structure of MoS_2_ particles, molybdenum disulfide particles coated with TiO_2_ nanoparticles (3–5 nm)	[33]
TiCl_4_, (NH_4_)_6_Mo_7_O_24_·4H_2_O, thiourea, ethanol, deionized water	hydrothermal treatment: 220 °C, reaction time: 24 h, drying: 60 °C for 12 h, calcination: 500 °C for 4 h	crystal structure characteristic for TiO_2_ (anatase) and MoS_2_, 8% loss of mass when heated to 700 °C, particles in the form of a single sheet, 20 nm TiO_2_ particles evenly distributed on the surface of MoS_2_	[34]
Ti(OC_4_H_9_)_4_, MoS_2_, ethylene glycol, HCl, deionized water	hydrothermal treatment: 180 °C, reaction time: 12 h	crystal structure of anatase and 2H-MoS_2_, BET surface area = 48.2 m^2^/g, MoS_2_ nanoparticles coated with TiO_2_ particles	[96]
Ti(OC_4_H_9_)_4_, Na_2_MoO_4_·2H_2_O, thiourea, HF, deionized water	preparation of TiO_2_ hydrothermal treatment: 180 °C, reaction time: 24 h preparation of TiO_2_-MoS_2_: hydrothermal treatment: 200 °C, reaction time: 24 h, drying: 105 °C for 24 h, calcination: 400 °C for 4 h	crystal structure of TiO_2_ (anatase) and MoS_2_ (for the molar ratio Mo:Ti = 7.5%), BET surface area = 86, 87, 98, 92 m^2^/g for systems obtained with molar ratios Mo:Ti = 2.5, 5, 7.5% respectively, TiO_2_ coated with MoS_2_ particles	[97]
TiO_2_-P25 (Degussa), Na_2_MoO_4_·2H_2_O, thiourea, HCl, NaOH, deionized water	preparation of TiO_2_: hydrothermal treatment: 180 °C, reaction time: 24 h, preparation of TiO_2_-MoS_2_: hydrothermal treatment: 180 °C, reaction time: 24 h, drying: 80 °C for 12 h	crystal structure characteristic for TiO_2_ and MoS_2_, MoS_2_ particles in the form of nanofibres with embedded spherical TiO_2_ agglomerates	[98]
TiCl_4_, MoS_2_, ethanol, glycerol, sodium hexametaphosphate	obtaining of TiO_2_: sonication preparation of TiO_2_-MoS_2_: hydrothermal treatment: 140 °C, reaction time: 3 h	crystal structure of TiO_2_ (anatase), M_3_O_8_·2H_2_O (ilsemanite) and 3R-MoS_2_ (molybdenite), particle diameter 46 nm, MoS_2_ particles coated with spherical TiO_2_ particles, 23% mass loss of the TiO_2_-MoS_2_ hybrid when heated to 900 °C	[99]
Ti, Na_2_MoO_4_·2H_2_O, thioacetamide, deionized water, acetone, ethanol	preparation of TiO_2_: anodic process on steel substrates in DSMO/HF electrolyte, calcination: 500 °C for 4 h preparation of TiO_2_-MoS_2_: hydrothermal treatment: 220 °C, reaction time: 24 h, drying: 80 °C for 12 h	crystal structure of anatase and hexagonal MoS_2_, particles in the form of nanotubes coated with MoS_2_	[100]
TiF_4_, Na_2_MoO_4_·2H_2_O, thioacetamide, deionized water	preparation of TiO_2_: hydrothermal treatment: 140 °C, reaction time: 1.5 h preparation of TiO_2_-MoS_2_: hydrothermal treatment: 220 °C, reaction time: 24 h, drying: 60 °C for 12 h	crystal structure of TiO_2_ (anatase) and rhombohedral MoS_2_, disappearance of anatase with an increase in the fraction of MoS_2_, TiO_2_ spheres covered with MoS_2_ nanoparticles	[101,102,103]
Ti(OC_4_H_9_)_4_, MoS_2_, H_2_SO_4_, *N*,*N*-dimethylformamide, ethanol, deionized water,	hydrothermal treatment: 200 °C, reaction time: 20 h	crystal structure of TiO_2_ (anatase) and MoS_2_, dense covering of molybdenum disulfide particles with titanium dioxide particles of 10 nm diameter	[104]
Ti(OC_4_H_9_)_4_, Na_2_MoO_4_·2H_2_O, thioacetamide, HF	hydrothermal treatment: 200 °C, reaction time: 24 h, drying: 80 °C for 12 h, calcination: 400 °C for 1 h	crystal structure of TiO_2_ (anatase) and MoS_2_, number of MoS_2_ layers in the range 6–9, TiO_2_ particles applied to MoS_2_ nanoparticles	[105]

**Table 4:** Resources, synthesis conditions and physico-chemical properties of TiO_2_-ZnO hybrid systems obtained by the sol-gel method.

Resources	Synthesis Conditions	Physico-Chemical Properties of the Final Product	References
TiCl_4_, Zn(CH_3_COO)_2_·2H_2_O, ZnCl_2_, benzyl alcohol, propan-2-ol, deionized water	ZnO sol: Zn(CH_3_COO)_2_·2H_2_O (sample A) or ZnCl_2_ (sample B), propan-2-ol TiO_2_-ZnO system: ZnO sol, TiCl_4_ mixed in the ratio TiO_2_:ZnO = 9:1 (sample A); 5:5 (sample B) calcination: 500 °C for 6 h (sample A); 200; 400; 550; 600 °C for 2 h (sample B)	crystal structure of anatase (sample A) and anatase, rutile and zinc titanate (sample B), spherical shaped particles with agglomeration	[1]
TiO_2_, Zn(CH_3_COO)_2_·2H_2_O, diethylamine, propan-2-ol, deionized water	TiO_2_ sol: TiO_2_ compact layer was deposited on an ITO (indium tin oxide) coated glass substrates by RF-sputtering technique with RF-power of 150 W, calcination: 450 °C for 30 min ZnO sol: Zn(CH_3_COO)_2_·2H_2_O, propan-2-ol TiO_2_-ZnO system: the TiO_2_ layer was immersed in ZnO sol solution	bands on XPS spectra—Ti2s, Ti3s, Ti2p, Zn2p, Zn3p, Zn3s, O1s, spherical particles, TiO_2_ particles coated with ZnO (core-shell)	[152]
Ti(OC_4_H_9_)_4_, Zn(CH_3_COO)_2_·2H_2_O, ethylamine, glacial acetic acid, ethanol, deionized water	TiO_2_ sol: Ti(OC_4_H_9_)_4_, ethanol, acetic acid ZnO sol: Zn(CH_3_COO)_2_·2H_2_O, ethanol, ethylamine, acetic acid TiO_2_-ZnO system: the sols of the respective oxides were mixed at molar ratio TiO_2_:ZnO = 7:3, 5:5, 3:7	antistatic properties, homogeneous surface of polyester material coated with a TiO_2_-ZnO oxide system	[153]
Ti(OC_4_H_9_)_4_, Zn(CH_3_COO)_2_·2H_2_O, glacial acetic acid, diethylamine, ethanol, deionized water	TiO_2_ sol: Ti(OC_4_H_9_)_4_, ethanol, acetic acid ZnO sol: Zn(CH_3_COO)_2_·2H_2_O, ethanol TiO_2_-ZnO system: obtained sols were mixed at molar ratio TiO_2_:ZnO = 1:0; 3:1; 1:3; 0:1, the obtained materials were deposited on carbon steel by immersion in solution, drying: 70 °C for 10 h calcination: 350, 500 °C for 2 h	crystal structure of Fe, TiO_2_, Fe_3_O_4_, ZnFe_2_O_4_, morphology – rough surfaces with numerous cracks, EDX spectrum showed bands characteristic for Ti, O, Fe, Zn	[154]
Ti(OC_4_H_9_)_4_, Zn(CH_3_COO)_2_·2H_2_O, hydrochloric acid, anhydrous ethanol, deionized water	TiO_2_ sol: Ti(OC_4_H_9_)_4_, ethanol, HCl ZnO sol: Zn(CH_3_COO)_2_·2H_2_O, ethanol, deionized water TiO_2_-ZnO system: materials obtained at molar ratio ZnO:TiO_2_ = 0.1:0.15; 0.2:0.25; 0.3:0.35, calcination: 450; 480; 500; 550; 600 °C for 1; 1.5; 2; 2.5; 3 h	crystal structure of anatase and wurtzite, particles with spherical shape with a tendency to agglomerate, BET surface area 76.258 m^2^/g, pore volume 0.0361 cm^3^/g, pore diameter 6.6 nm	[155]
Ti(OC_4_H_9_)_4_, Zn(CH_3_COO)_2_·2H_2_O, nitric acid, ethylene glycol, glycerol, trimethylamine (TEA), anhydrous ethanol, deionized water	TiO_2_ sol: Ti(OC_4_H_9_)_4_, nitric acid, ethanol ZnO sol: Zn(CH_3_COO)_2_·2H_2_O, ethylene glycol, glycerol, TEA, ethanol TiO_2_-ZnO system: obtained sols were mixed at molar ratio TiO_2_:ZnO = 100:0, 75:25, 50:50, 25:75, 0:100, calcination: 500 °C for 1 h	anatase crystal structure (TiO_2_:ZnO = 100:0, 75:25), amorphous structure (TiO_2_:ZnO = 50:50, 25:75), wurtzite structure (TiO_2_:ZnO = 0:100), XPS shows the presence of Ti2p, Zn2p, O1s bands	[156]
Ti(OC_4_H_9_)_4_, Zn(CH_3_COO)_2_·2H_2_O, nitric acid, acetic acid, diethylamine, acetylacetone, anhydrous ethanol, deionized water	TiO_2_ sol: Ti(OC_4_H_9_)_4_, ethanol, acetylacetone, acetic acid, diethylamine ZnO sol: Zn(CH_3_COO)_2_·2H_2_O, ethanol, diethylamine TiO_2_-ZnO system: obtained sols were mixed at molar ratio TiO_2_:ZnO = 90:10, 80:20, 70:30, 60:40, calcination: 500 °C for 30 min	anatase crystal structure (TiO_2_:ZnO = 90:10, 80:20), amorphous structure (TiO_2_:ZnO = 70:30, 60:40), spherical shaped particles and porous structure, XPS shows bands characteristic for Ti2p, Zn2p, O1s	[157]
Ti[OCH(CH_3_)_2_]_4_, Zn(NO_3_)_2_·6H_2_O, glacial acetic acid, ethanol, deionized water	TiO_2_ sol: Ti(OC_4_H_9_)_4_, ethanol ZnO sol: Zn(NO_3_)_2_·6H_2_O, ethanol, glacial acetic acid TiO_2_-ZnO: obtained sols were mixed at molar ratio TiO_2_:ZnO = 10:3, calcination: 400 °C for 2 h	crystal structure of anatase and zincite, particles with spherical shape and a tendency to agglomerate, EDX shows the presence of bands derived from Ti, Zn, O	[158]
Ti(OC_4_H_9_)_4_, Zn(NO_3_)_2_·6H_2_O, citric acid, deionized water	TiO_2_-ZnO system: sol-gel auto-ignition method, citric acid (catalyst) Ti(OC_4_H_9_)_4_, Zn(NO_3_)_2_·6H_2_O was heated to a temperature of 300 °C, auto-ignition, calcination: 500 °C for 5 h	crystal structure of anatase, BET surface area = 52, 63.4, 69.9, 64, 36.6 m^2^/g respectively for systems prepared with a ZnO mass fraction of 1, 5, 10, 12, 30%, particles of irregular shape with a tendency to agglomerate, EDX shows bands characteristic for Ti, O, Zn	[159]
Ti[OCH(CH_3_)_2_]_4_, Zn(CH_3_COO)_2_·2H_2_O, acetic acid, diethylamine, butanol, propan-2-ol, deionized water	TiO_2_ sol: Ti(OC_4_H_9_)_4,_ butanol, acetic acid ZnO sol: Zn(CH_3_COO)_2_·2H_2_O, propan-2-ol, diethylamine, deionized water TiO_2_-ZnO system: the glass plate was covered with a layer of TiO_2_ sol, calcination: 400 °C for 2 h, covering by ZnO layer, calcination: 500, 600 °C for 2 h	crystal structure of anatase and wurtzite, increase of surface roughness with increasing calcination temperature	[160]
Ti(OC_4_H_9_)_4_, Zn(CH_3_COO)_2_·2H_2_O, sodium hydroxide, propan-2ol, deionized water	sol of TiO_2_: Ti(OC_4_H_9_)_4_, propan-2-ol sol of ZnO: Zn(CH_3_COO)_2_·2H_2_O, sodium hydroxide (0.5 M), deionized water TiO_2_-ZnO system: obtained sols was mixed at molar ratio TiO_2_:ZnO = 0.5:0.25; 1:0.5; 1.5:0.75; 2:1, are designated respectively as TZO1 TZO2, TZO3 and TZO4, calcination: 550 °C for 4 h	crystalline structure of anatase and wurtzite, Raman spectroscopy - bands characteristic for TiO_2_ and ZnO, spherical shaped particles and rods with tendency to agglomerate, EDX—strands derived from Ti, Zn, O, BET surface area equal to 74.7, 32.2, 12.58. 8.82 m^2^/g respectively for samples TZO1 TZO2, TZO3, and TZO4	[161]
Ti(OC_4_H_9_)_4_, Zn(CH_3_COO)_2_·2H_2_O, nitric acid, ethylene glycol, ethanol, deionized water	TiO_2_ sol: Ti(OC_4_′H_9_)_4_, ethanol, nitric acid ZnO sol: Zn(CH_3_COO)_2_·2H_2_O, ethylene glycol TiO_2_-ZnO system: TiO_2_ and ZnO sols were mixed at molar ratio TiO_2_:ZnO = 2:1, calcination: 450 °C for 1 h	particles of spherical shape with no tendency to agglomerate, EDX showed characteristic bands for Ti, Zn, O, energy band gap 3.41 eV	[162]
Ti(OC_4_H_9_)_4_, Zn(CH_3_COO)_2_·2H_2_O, nitric acid, diethylamine, acetylacetone, propan-2-ol, deionized water	TiO_2_ sol: Ti(OC_4_H_9_)_4_, acetylacetone, propan-2-ol, nitric acid ZnO sol: Zn(CH_3_COO)_2_·2H_2_O, propan-2-ol, diethylamine TiO_2_-ZnO system: obtained sols were mixed in equimolar ratio	irregular shaped particles, EDX showed bands characteristic for Ti, Zn, O	[163]
TiCl_4_, Zn(CH_3_COO)_2_·2H_2_O, ammonium fluoride, acetonitrile, butanol, acetone, diethylamine, ethanol, deionized water	TiO_2_: electrode method ZnO sol: Zn(CH_3_COO)_2_·2H_2_O, ethanol diethylamine TiO_2_-ZnO system: TiO_2_ (anodic method) was immersed in a solution of TiCl_4_, calcination: 450 °C for 15 min, TiO_2_ was covered by ZnO layer, drying: 180 °C for 10 min, calcination: 500 °C for 1 h	particles with shapes similar to nanotubes and spherical, EDX showed characteristic bands for Ti, Zn, O	[164]
Ti[OCH(CH_3_)_2_]_4_, Zn(CH_3_COO)_2_·2H_2_O, acetic acid, propan-2-ol	TiO_2_-ZnO system: to Ti[OCH(CH_3_)_2_]_4_ was added Zn(CH_3_COO)_2_·2H_2_O dissolved in propan-2-ol (molar ratio Ti:Zn = 5:1), calcination: 400 °C for 4 h	crystal structure of anatase and wurtzite, particles with a spherical shape with agglomeration, BET surface area = 91 m^2^/g, pore size 1.49 nm, pore volume 0.343 cm^3^/g	[165]

**Table 5:** Resources, synthesis conditions and physico-chemical properties of TiO_2_-ZrO_2_ oxide systems obtained by the sol-gel method.

Resources	Synthesis Conditions	Physico-Chemical Properties of the Final Product	References
Ti(OC_4_H_9_)_4_, ZrOCl_2_·8H_2_O, nitric acid, polyethylene glycol (PEG), (PEO)_20_(PPO)_70_(PEO)_20_ (Pluronic P123, M_W_ = 5800, Aldrich), ethanol, deionized water	TiO_2_-ZrO_2_ oxide system: single-step synthesis, mixed with Ti(C_4_H_9_)_4_: PEG:P123 with ZrOCl_2_·8H_2_O (molar ratio Ti:Zr = 1:0.1), aging: 24 h, calcination: 800 °C for 5 h	crystal structure of anatase, tetragonal zirconia rutile, BET surface area = 148.9; 138.5; 136.9 m^2^/g for TiO_2_-ZrO_2_(P123 + PEG); TiO_2_-ZrO_2_(PEG); TiO_2_-ZrO_2_(P123)	[29]
Ti[OCH(CH_3_)_2_]_4_, Zr[OCH(CH_3_)_2_]_4_, nitric acid, deionized water	TiO_2_-ZrO_2_ oxide systems: M1 and M2 M1: Ti_0.9_Zr_0.1_O_2_, hydrolysis of precursors in an aqueous environment M2: Ti_0.9_Zr_0.1_O_2_, polymerization of precursors in propan-2-ol drying: 100 °C, calcination: 350 °C for 2 h	crystal structure of anatase and brookite, Raman spectroscopy showed anatase-specific bands, BET surface area = 313 m^2^/g (M1); 269 m^2^/g (M2)	[188]
Ti[OCH(CH_3_)_2_]_4_, Zr[OCH(CH_3_)_2_]_4_, ammonia water, propan-2-ol, distilled water	TiO_2_ sol: Ti[OCH(CH_3_)_2_]_4_, propan-2-ol, ammonia water ZrO_2_ sol: Zr[OCH(CH_3_)_2_]_4_, propan-2-ol, ammonia water TiO_2_-ZrO_2_ oxide systems: sol was mixed with ZrO_2_ in a molar fraction of 3%, 6%, 13%, 37%, drying: 100 °C for 24 h, calcination: 550, 700 °C for 5 h	crystal structure of anatase and rutile, spherical particle shape with agglomeration, BET surface area = 26, 37, 40, 172 m^2^/g for materials calcined at 550 °C and 26, 29, 30, 36 m^2^/g for materials calcined at 700 °C, for systems with ZrO_2_ molar fractions 3%, 6%, 13%, 37% respectively	[189]
Ti[OCH(CH_3_)_2_]_4_, Zr(CH_3_COO)_2_·2H_2_O, hydrochloric acid, hydroxypropylcellulose, deionized water	ZrO_2_ sol: Zr(CH_3_COO)_2_·2H_2_O, hydroxypropylcellulose TiO_2_ sol: Ti[OCH(CH_3_)_2_]_4_, HCl, water TiO_2_-ZrO_2_: the sol was mixed maintaining the ratio Ti:Zr = 0.5:0.5, drying: 150 °C for 1 h, calcination: 300, 500, 700, 900 °C for 1 h	crystal structure of anatase and zirconium dioxide, crystallite size 5.8 and 8.5 nm for calcination temperatures of 500 °C and 900 °C, particles with a spherical shape with a tendency to agglomerate	[190]
Ti[OCH(CH_3_)_2_]_4_, Zr[OCH(CH_3_)_2_]_4_, poly(ethylene oxide), (PEO)_20_(PPO)_70_(PEO)_20_ (Pluronic P123, BASF), acetylacetone, hydrochloric acid, deionized water	TiO_2_ sol: Ti[OCH(CH_3_)_2_]_4_, HCl, water ZrO_2_ sol: Zr[OCH(CH_3_)_2_]_4_, HCl, water TiO_2_-ZrO_2_: the sol was mixed, aging: 12 h, calcination: 400, 500 °C for 2 h	crystal structure of anatase, incorporation of zirconium dioxide into titania network forming Ti_1−x_Zr_x_O_2_ with anatase crystal structure, Raman spectroscopy showed anatase and ZrO_2_ characteristic bands, spherical shaped particles densely packed with diameter 10–15 nm	[191]
Ti[OCH(CH_3_)_2_]_4_, Zr[OCH(CH_3_)_2_]_4_, lauramine hydrochloride, acetyl acetone, diethylamine, deionized water	TiO_2_-ZrO_2_ oxide system: single-step synthesis, mixed Ti[OCH(CH_3_)_2_]_4_, Zr[OCH(CH_3_)_2_]_4_ acetylacetone and laurylamine, aging: 7 days, calcination: 500–900 °C	crystal structure of anatase, rutile and tetragonal zirconia, particles with spherical shape with agglomeration	[27]
Ti[OCH(CH_3_)_2_]_4_, Zr[OCH(CH_3_)_2_]_4_, nitric acid, ethanol, deionized water	TiO_2_-ZrO_2_: one-stage synthesis, hydrolysis of Ti[OCH(CH_3_)_2_]_4_, Zr[OCH(CH_3_)_2_]_4_ in ethanol:water	Fourier-transform infrared spectroscopy (FTIR) spectroscopy showed bands characteristic for TiO_2_ and ZrO_2_, distances between planes 3.533 Å	[192]
Ti[OCH(CH_3_)_2_]_4_, Zr[OCH(CH_3_)_2_]_4_, diethylamine (DEA), isoeugenol (ISOH), propan-1-ol, deionized water	TiO_2_-ZrO_2_ oxide system: one-stage synthesis, Ti[OCH(CH_3_)_2_]_4_, Zr[OCH(CH_3_)_2_]_4_ mixed with a chelating complex (diethylamine or isoeugenol), calcination: 500, 650 °C in air and 350, 400 °C in a nitrogen atmosphere	amorphous structure (materials calcined at 500 °C), crystal structure of anatase and rhombic TiO_2_-ZrO_2_ (materials calcined at 650 °C), BET surface area = 7.8 m^2^/g (TiO_2_-ZrO_2_-ISOH) and 8.3 m^2^/g (TiO_2_-ZrO_2_-DEA) for samples calcined in a nitrogen atmosphere, 240 m^2^/g (TiO_2_-ZrO_2_-ISOH) and 186 m^2^/g (TiO_2_-ZrO_2_-DEA) for samples calcined in air	[193]
TiF_4_, Zr(NO_3_)_4_, anodic aluminium oxide (AAO), ethanol, sulphuric acid, deionized water	TiO_2_-ZrO_2_ oxide system: single-step synthesis, AAO was immersed in Zr(NO_3_)_4_, drying: 80 °C for 1 h, immersed in TiF_4_ for 9 min, calcination: 600 °C for 3 h	crystal structure of anatase and monoclinic zirconia, nanotube-shaped particles densely packed, BET surface area = 47.4 m^2^/g	[26]
Ti(OC_4_H_9_)_4_, Zr(OC_4_H_9_)_4_, butan-1-ol, ammonium hydroxide, deionized water	TiO_2_-ZrO_2_ oxide system: single-step synthesis, hydrolysis of Ti(OC_4_H_9_)_4_, Zr(OC_4_H_9_)_4_ in butan-1-ol:water, ammonium hydroxide was added to neutralize the pH, drying: 120 °C for 12 h, calcination: 500 °C for 12 h	crystal structure of anatase, monoclinic and tetragonal zirconia, Raman spectroscopy showed anatase-specific bands, BET surface area = 68, 62 and 60 m^2^/g for systems obtained at molar ratio TiO_2_:ZrO_2_ = 3:1, 1:1, 1:3	[194]
Ti[OCH(CH_3_)_2_]_4_, Zr[OCH(CH_3_)_2_]_4_, ethanol, deionized water	TiO_2_-ZrO_2_: single-step synthesis, hydrolysis of Ti(OC_4_H_9_)_4_, Zr(OC_4_H_9_)_4_ in ethanol:water, drying: 100 °C for 6 h, calcination: 550 °C for 2 h	crystal structure of anatase, monoclinic and tetragonal zirconia, spherical shaped particles with a pronounced tendency to agglomerate, BET surface area 32.5, 42.5, 36 m^2^/g for 67%TiO_2_-33%ZrO_2_, 50%TiO_2_-50%ZrO_2_, 33%TiO_2_-67%ZrO_2_	[195]

**Table 6:** Process parameters affecting the electrospinning method.

Process Parameters		
Solution	Apparatus	External Environment
type of solvent	applied voltage	temperature
concentration	distance of nozzle from collector	humidity
-	diameter and length of nozzle	-

**Table 7:** Resources, synthesis conditions and physico-chemical properties of TiO_2_-ZnO hybrid systems obtained by the electrospinning method.

Resources	Synthesis Conditions	Physico-Chemical Properties of the Final Product	References
Ti[OCH(CH_3_)_2_]_4_, Zn(CH_3_COO)_2_·2H_2_O, polyvinylpyrrolidone, acetic acid, ethanol, deionized water	solution of TiO_2_ and ZnO: Zn(CH_3_COO)_2_·2H_2_O, acetic acid and Ti[OCH(CH_3_)_2_]_4_ dissolved in a mixture of ethanol and polyvinylpyrrolidone TiO_2_-ZnO electrospinning: voltage: +7 kV, spinning speed 0.3 cm^3^/h calcination: 500 °C for 3 h	nanofibre shaped particles, X-ray photoelectron spectroscopy (XPS) shows bands derived from Ti2p, O1s, Zn2p	[232]
Ti[OCH(CH_3_)_2_]_4_, Zn(C_2_H_5_)_2_, dimethylformamide (DMF), acetic acid, poly(vinyl acetate), deionized water	preparation of TiO_2_ solution: Ti[OCH(CH_3_)_2_]_4_, acetic acid, dimethylformamide, polyvinyl acetate TiO_2_ electrospinning: voltage: +10 kV, spinning speed 0.2 cm^3^/h, calcination: 600 °C for 8 h TiO_2_-ZnO oxide system: ZnO was applied by atomic layer deposition (ALD)	crystal structure of anatase and wurtzite, nanofibre particles of core-shell structure, average nanofibre diameter 250 nm, increase of ZnO coating thickness 0.66 nm per ALD cycle	[233]
Ti[OCH(CH_3_)_2_]_4_, ZnCl_2_, polyvinylpyrrolidone, (PEO)_20_(PPO)_70_(PEO)_20_ (Pluronic P123), ethanol, deionized water	preparation of TiO_2_ and ZnO precursors: polyvinylpyrrolidone dissolved in ethanol, Ti(OCH(CH_3_)_2_)_4_ solution added to aqueous ZnCl_2_ solution, calcination: 500 °C for 4 h TiO_2_-ZnO electrospinning: voltage: +20 kV	crystal structure of anatase, wurtzite and zinc titanate (ZnTiO_3_), particles with diameter 20 nm, BET specific surface area = 203 m^2^/g, pore volume 0.24 cm^3^/g	[234]
Ti[OCH(CH_3_)_2_]_4_, Zn(CH_3_COO)_2_·2H_2_O, cellulose acetate, dimethylformamide (DMF), acetic acid, acetone, deionized water	preparation of TiO_2_ and ZnO precursors: Ti[OCH(CH_3_)_2_]_4_, Zn(CH_3_COO)_2_·2H_2_O cellulose acetate was dissolved in a mixture of DMF:acetone = 1:2 (*v*/*v*) TiO_2_-ZnO electrospinning: voltage: +10 kV, spinning speed 10 μL/min, calcination: 500 °C for 5 h	crystal structure of anatase, wurtzite and zinc titanate (ZnTiO_3_), diameter of nanofibres 85–200 nm, cylindrical nanofibres made of granular nanoparticles, XPS showed characteristic bands Ti2p, O1s and Zn2p	[235]
Ti[OCH(CH_3_)_2_]_4_, ZnO, poly(vinyl acetate) (PVA), dimethylformamide (DMF), acetic acid, deionized water	preparation of TiO_2_ and ZnO precursors: polyvinyl acetate was dissolved in DMF, added Ti[OCH(CH_3_)_2_]_4_ and ZnO TiO_2_-ZnO electrospinning: voltage: +12 kV, drying: 80 °C for 12 h, calcination: 600 °C for 1 h	crystal structure of wurtzite, nanofibres with a smooth surface, EDX showed presence of elements Ti, Zn, O	[236]
Ti[OCH(CH_3_)_2_]_4_, Zn(CH_3_COO)_2_·2H_2_O, polyvinylpyrrolidone, dimethylformamide (DMF), ethanol, deionized water	preparation of precursors TiO_2_ and ZnO: Ti[OCH(CH_3_)_2_]_4_ and Zn(CH_3_COO)_2_·2H_2_O dissolved in DMF, polyvinylpyrrolidone was added TiO_2_-ZnO electrospinning: voltage: +10 kV, calcination: 400 °C for 2 h	crystal structure of anatase and wurtzite, nanofibres composed of individual nanoparticles, XPS showed presence of specific bands for Ti2p, Zn2p, O1s	[237]
TiO_2_ (P25), Zn(CH_3_COO)_2_·2H_2_O, hexamethyltetramine, ethanol, demineralized water	electrospinning of TiO_2_ fibres: P25 solution and polymer in ethanol, applied voltage: +15 eV, TiO_2_ fibres mixed with solution 1 heating: 120 °C for 1 h TiO_2_ nanofibres were immersed in solution 2 (aqueous zinc acetate solution and hexamethyl tetramine) and maintained at 85 °C for 24 h, calcination: 500 °C for 1 h	crystal structure of anatase, rutile and wurtzite, TiO_2_ fibres coated with ZnO nanorods, EDX spectrum contained specific bands characteristic for Ti, O, Zn, Raman spectrum contained bands characteristic for TiO_2_, ZnO and ZnTiO_3_	[238]

**Table 8:** Resources, synthesis conditions and physico-chemical properties of TiO_2_-ZrO_2_ oxide systems obtained by the electrospinning method.

Resources	Synthesis Conditions	Physico-Chemical Properties of the Final Product	References
Ti(O_4_H_9_)_4_, ZrOCl_2_, polyvinylpyrrolidone, acetic acid, ethanol, deionized water	solution of ZrO_2_:ZrOCl_2_ was dissolved in ethanol:water, poly(vinylpyrrolidone) was added, stirred for 12 h at room temperature TiO_2_:Ti(O_4_H_9_)_4_ solution was hydrolysed in ethanol:acetic acid, polyvinylpyrrolidone solution was added, stirred for 6 h at room temperature TiO_2_-ZrO_2_ electrospinning: voltage for the ZrO_2_ stream: +5 kV, voltage for TiO_2_: −7 kV, calcination: 600 °C for 3 h	crystal structure of anatase and tetragonal zirconia, presence of nanofibers with different diameters, (130 nm and 70 nm for ZrO_2_ and TiO_2_ respectively)	[254]
Ti[OCH(CH_3_)_2_]_4_, Zr[OCH(CH_3_)_2_]_4_, polyvinylpyrrolidone, phosphoric acid, acetic acid, ethanol, deionized water	TiO_2_ and ZrO_2_ precursor solution: polyvinylpyrrolidone dissolved in ethanol, mixed for 1 h, acetic acid, Ti [CH(CH_3_)_2_]_4_, Zr[OCH(CH_3_)_2_]_4_ (atomic ratio Ti:Zr = 1:1) TiO_2_-ZrO_2_ electrospinning: voltage 14 kV, spinning speed 30 μL/min, calcination: 500, 700, 1000 °C for 6 h	amorphous structure for material calcined at 500 °C, crystal structure of srilankite (Ti_0.5_Zr_0_._5_O_2_) for calcination temperature 700 and 1000 °C, BET surface area = 75 m^2^/g for material calcined at 500 °C, average fibre diameter 497 nm (before calcination) and 344 nm (after calcination at 500 °C)	[255]
